# ACSL5 programs fatty acid metabolism and mitochondrial fitness to sustain pathogenic T cells and exacerbate Sjögren's syndrome

**DOI:** 10.7150/ijbs.131033

**Published:** 2026-07-13

**Authors:** Xinyi Ren, Junhao Yin, Xinyi Ma, Jiabao Xu, Changyu Chen, Lele He, Ruowen Zhao, Yucheng Xu, Yiping Pu, Baoli Wang, Jiayao Fu, Lingyan Zheng

**Affiliations:** 1 Department of Oral Surgery, Shanghai Ninth People's Hospital, College of Stomatology, Shanghai Jiao Tong University School of Medicine, Shanghai 200001, China.; 2 National Center for Stomatology & National Clinical Research Center of Oral Disease, Shanghai Key Laboratory of Stomatology, Shanghai 200001, China.; 3 Shanghai Engineering Research Center of Tooth Restoration and Regeneration & Tongji Research Institute of Stomatology & Department of Prosthodontics, Shanghai Tongji Stomatological Hospital and Dental School, Tongji University, Shanghai 200072, China.; 4 Würzburg Institute of Systems Immunology, Max Planck Research Group, Julius-Maximilians University of Würzburg, Würzburg 97255, Germany.; 5 Shanghai Key Laboratory of Craniomaxillofacial Development and Diseases, Shanghai Stomatological Hospital and School of Stomatology, Fudan University, Shanghai 200001, China.

**Keywords:** Sjögren's syndrome, T cells, ACSL5, fatty acid oxidation, MERCs, T-cell memory

## Abstract

Emerging evidence has shown that fatty acid metabolism is closely associated with autoreactive T cells in autoimmunity, but its function in Sjögren's syndrome (SS) is still unclear. Here, we identified acyl-CoA synthetase long-chain family member 5 (ACSL5) as a metabolic checkpoint that drives pathogenic T-cell responses in SS. ACSL5 was upregulated in patients with SS and positively correlated with T-cell infiltration and lipid dysregulation. ACSL5-high T cells presented hyperactive effector activity and a proinflammatory phenotype. Metabolic profiling indicated that ACSL5 increased fatty acid uptake and utilization and promoted fatty acid oxidation (FAO) through peroxisome proliferator-activated receptor alpha (PPARα) in T cells, thereby improving mitochondrial respiratory capacity. Mechanistically, ACSL5 facilitated the nuclear translocation of PPARα and subsequent Mitofusin 2 (*MFN2*) transcription, increasing mitochondrial elongation and the formation of mitochondria‒endoplasmic reticulum contacts (MERCs) to influence the FAO and T-cell response. Disruption of the ACSL5/PPARα/MFN2 axis attenuated effector functions and reduced the longevity of pathogenic effector T cells. Pharmacological inhibition of FAO or ACSL5 decreased inflammatory T-cell infiltration and alleviated salivary gland inflammation. Collectively, these findings reveal an ACSL5-centered metabolic program that sustains pathogenic T-cell responses in SS and suggest ACSL5/FAO as a potential therapeutic target.

## Introduction

Sjögren's syndrome (SS) is a chronic autoimmune disorder primarily involving the salivary and lacrimal glands 1, whose pathogenesis is complex and remains to be fully elucidated 2. T cells play a central role in the pathogenesis of SS, as indicated by the predominance of lymphocyte infiltration (>75%) 3 and significant clonal expansion (including CD4^+^ and CD8^+^ T cells) 4. Various effector T-cell subsets, including T helper 1 (Th1), T helper 17 (Th17), and follicular helper T (Tfh) cells, contribute to disease progression, whereas regulatory T (Treg) cells, which normally exert suppressive effects on autoimmune responses, exhibit reduced proportions and impaired function in patients with SS 5. Therefore, targeting pathogenic T-cell responses represents a promising therapeutic strategy for treating SS.

Upon activation, T cells require substantial amounts of energy and protein substrates to support their clonal expansion and immune responses 6. Accordingly, metabolic reprogramming plays a pivotal role in T-cell activation during autoimmune diseases 7. Among various metabolic pathways, fatty acid (FA) metabolism, including fatty acid β-oxidation (FAO), has been recently shown to regulate T-cell function. FAO is initiated by carnitine palmitoyltransferase 1A (CPT1A) 8 and channeled by acyl-coenzyme A synthetase long-chain family members (ACSLs) 9, which convert long-chain fatty acids into acyl-CoAs to generate ATP for T-cell energy and longevity 10. The ACSL family comprises five members (ACSL1, ACSL3, ACSL4, ACSL5, and ACSL6), each with distinct subcellular localization, tissue distribution, and substrate preferences 11. However, the expression profiles of different ACSL members in T cells are not uniform, and their catalytic preferences for fatty acid products (e.g., oleic acid, linoleic acid, palmitic acid, and palmitoleic acid) also vary 12. Thus, elucidating the specific roles of fatty acid metabolic regulators in T cells is critical for developing precision therapies for SS.

In this study, we identified ACSL5 as a key FAO-catalyzing enzyme that is preferentially upregulated in activated T cells from patients with SS. Unlike other ACSL members, ACSL5 is localized primarily to the outer mitochondrial membrane and has high catalytic activity toward long-chain fatty acids 13. Notably, ACSL5 expression is elevated in T-cell-enriched tissues, including the spleen and lymph nodes, suggesting its potential involvement in immune responses 14. However, whether and how ACSL5 regulates T-cell immunity remains largely unexplored. Here, we systematically investigate the role and underlying molecular mechanisms of ACSL5, establishing it as a critical regulator of T-cell metabolic fitness and effector function, and further show that ACSL5 preferentially sustains inflammatory T-cell subsets, thus highlighting ACSL5/FAO as a promising therapeutic target for the treatment of SS and related autoimmune diseases.

## Materials and Methods

### Bioinformatic analysis

A total of 2591 metabolism-related genes (MRGs) were derived from the Molecular Signatures Database 15, GeneCards, and Biocarta. Minor salivary gland (MSG) biopsy datasets were retrieved from GEO (GSE23117 16, GSE127952 17, GSE40611, and GSE80805). The raw GEO data were standardized and normalized. DEGs were identified and intersected with metabolism-related genes (MRGs) to identify DEMRGs. Protein‒protein interaction (PPI), pathway enrichment and immune infiltration analyses were performed to further identify the correlation between DEMRGs and the pathogenesis of SS. Correlations were calculated via Pearson's coefficient.

The single-cell datasets GSE179623 18 (CD4^+^ T cells) and GSE272409 19 (SS salivary glands) were analyzed via Seurat 20. After quality control, uniform manifold approximation and projection (UMAP)/t-distributed stochastic neighbor embedding (t-SNE) clustering and annotation (SingleR, canonical markers) were performed. DEGs were identified via gene set enrichment analysis (GSEA), pseudotime (Monocle2), metabolic activity (scMetabolism 21, AUCell), and stemness (CytoTRACE 22) analyses. Module scores were calculated to assess lineage-associated programs, and transcription factor activity was inferred using DoRothEA with decoupleR. Lineage dynamics were modeled using generalized additive models.

### Primary T-cell isolation and cell culture

Primary mouse CD4^+^ and CD8^+^ T cells were isolated from the spleen (STEMCELL Technologies; Vancouver, BC, Canada; Cat# 18952/19753) and stimulated for 72 h with anti-CD3 (1 μg/mL; BioLegend, San Diego, CA, USA; Cat# 100301) and anti-CD28 antibodies (2 μg/mL; BioLegend; Cat# 102101). T-cell differentiation assays were conducted as previously described 23. Human Jurkat T cells (Cell Bank of China, Shanghai, China) were cultured in RPMI-1640 (10% fetal bovine serum, 1% penicillin/streptomycin). To activate the Jurkat T cells, the cells were plated in 6-well plates precoated with anti-CD3 (1 μg/mL; Thermo Fisher, Cat# 16-0037-81) and anti-CD28 (2 μg/mL; Thermo Fisher, Cat# 16-0281-82) antibodies.

### Animal models

Female NOD/Ltj mice (8 weeks old; GemPharmatech, Nanjing, China) under specific pathogen-free (SPF) conditions received intraperitoneal injections of etomoxir (15 mg/kg; Selleck, Houston, TX, USA; Cat# S8244), Triacsin C (15 mg/kg; MedChemExpress, Monmouth Junction, NJ, USA; Cat# HY-N6707), or the solvent control twice weekly for one month.

For adoptive transfer, 1 x 10^6^ sgRNA-guided *Acsl5*-knockout or wild-type (WT) OT-II CD4^+^ T cells were intravenously transferred into 12-week-old Rag1^-/-^ recipients. Twenty-four hours post-transfer, the mice were immunized with 50 µg of OVA protein. Draining lymph nodes were analyzed 14 days post-transfer. Recipient mice were immunized with 50 μg of OVA protein to induce antigen-driven T-cell activation and establish an experimental Sjögren's syndrome (ESS)-like autoimmune response, as described in previous studies 24.

### Ethics statement

All experiments involving animals were performed in compliance with relevant laws and institutional guidelines 25 and were approved by the Ethics Committee of Shanghai Jiao Tong University School of Medicine (Approval No. SH9H-2023-A401-SB). Research involving human subjects was conducted in accordance with the principles of the Declaration of Helsinki and was approved by the Ethics Committee of Shanghai Ninth People's Hospital (Approval No. SH9H-2019-T159-4).

### Quantitative real-time polymerase chain reaction (qRT‒PCR)

RNA extraction, reverse transcription, and quantification were performed as previously described 24. Gene expression levels were calculated using the comparative threshold cycle (Ct) method, with β-actin serving as an internal control (primers: Table 1).

### Western blotting

Proteins were extracted with radioimmunoprecipitation assay (RIPA) buffer (Sigma‒Aldrich, St. Louis, MO, USA; Cat# R0278) supplemented with a phosphatase inhibitor (Thermo Fisher Scientific, Waltham, MA, USA; Cat# 78441). Proteins were separated, transferred to polyvinylidene fluoride (PVDF) membranes, blocked (bovine serum albumin, BSA), and incubated with primary antibodies (4°C, overnight), followed by incubation with horseradish peroxidase (HRP)-conjugated secondary antibodies (Cell Signaling Technology, Danvers, MA, USA; Cat# 8114P; 2 h, room temperature). Bands were visualized (Millipore, Burlington, MA, USA) and quantified (ImageJ). Detailed information concerning the primary antibodies is provided in Table S1.

### Chromatin immunoprecipitation (ChIP) assay

The ChIP method used was previously described by Zhang *et al*. 26 (Cell Signaling Technology, Danvers, MA, USA; Cat# 90045) per the manufacturer's protocol. Purified ChIP DNA was quantified via qPCR (primers: Table 1) and normalized to the input.

### *In vitro* transfection

For mouse *Acsl5* knockout, retroviral CRISPR plasmids (Genechem, Shanghai, China) were packaged (Takara Bio, Kusatsu, Japan; Cat# 6160) and used to transduce primary T cells (primers: Table 2). Jurkat T cells were transduced with *ACSL5*-knockdown or scrambled lentivirus (Genechem, Shanghai, China) and selected with puromycin (2 μg/ml; Beyotime Biotechnology, Shanghai, China; Cat# ST551) for 48 h (primers: Table 3). Primary T cells were transfected with siRNAs (Genechem, Shanghai, China) via Lipofectamine 3000 (Invitrogen, Carlsbad, CA, USA; Cat# L3000001). The medium was replaced at 24 h, and the cells were collected at 48 h post-transfection (primers: Table 4).

### Cell Counting Kit-8 (CCK-8) assay

CCK-8 (Beyotime Biotechnology, Shanghai, China; Cat# C0038) was performed according to the manufacturer's protocol, following an approach similar to that of Yuan *et al*. 27.

### Flow cytometry

The cells were stained with surface antibodies (BioLegend, San Diego, CA, USA). For intracellular cytokine staining, the cells were restimulated (Cell Activation Cocktail, BioLegend; Cat# 423303; 6 h), fixed/permeabilized (eBioscience, San Diego, CA, USA; Cat# 004222) and stained (BioLegend). Data were acquired on a CytoFlex S (Beckman Coulter, Brea, CA, USA). Detailed information concerning the flow cytometry antibodies is provided in Table 2.

### Lipid staining and lipid peroxidation analysis

Intracellular lipid accumulation was assessed using Nile Red (Beyotime; Cat# C2051) and BODIPY 500/510 (Beyotime; Cat# C2055) according to the manufacturer's instructions. Lipid peroxidation was evaluated using BODIPY 581/591 C11 (Beyotime; Cat# S0043). The fluorescence intensity was analyzed by flow cytometry.

### Cell cycle analysis

The cell cycle distribution was determined using a PI cell cycle kit (Beyotime; Cat# C1052) following the manufacturer's protocol. Data were acquired by flow cytometry.

### Cell proliferation assay

Cell proliferation was assessed by EdU incorporation (Beyotime; Cat# C0071), Ki-67 expression (BioLegend; Cat# 652410), and CFSE dilution (Thermo Fisher Scientific; Cat# C34554) following standard protocols. Flow cytometry was used to quantify proliferative capacity.

### Apoptosis analysis

Apoptosis was assessed using an Annexin V-PE/7-AAD apoptosis detection kit (Yeasen Biotechnology, Shanghai, China; Cat# 40310ES20) following the manufacturer's instructions. Early and late apoptotic cells were analyzed by flow cytometry.

### Mitochondrial function analysis

The mitochondrial membrane potential was measured using JC-1 (Beyotime; Cat# C2006) or TMRE (Beyotime; Cat# C2001) according to the manufacturer's instructions. Mitochondrial mass was assessed using MitoTracker (Beyotime; Cat# C1049B). Mitochondrial ROS levels were detected using MitoSOX Red (Beyotime; Cat# S0061S), while intracellular ROS levels were measured using DCFH-DA (Beyotime; Cat# S0033). The fluorescence intensity was quantified by flow cytometry.

### Enzyme-Linked Immunosorbent Assay (ELISA)

Circulating blood from mice (0.5 mL) was obtained by retro-orbital bleeding, collected in a clot activator tube and isolated by centrifugation (1500 × g, 15 min, 4°C). Serum IL-17A (Abcam, Cambridge, UK; Cat# ab100702) and IFN-γ (Abcam; Cat# ab252363) levels were quantified via ELISA per the manufacturer's instructions 28. Anti-SSA and anti-SSB autoantibodies were measured using ELISA kits (FineTest; Cat# EM2223, Cat# EM2224).

### Histology and immunofluorescence

Submandibular salivary gland tissues were isolated from NOD/Ltj mice, fixed in 4% paraformaldehyde (PFA) for 24 hours at 4°C, and embedded in paraffin. The sections were stained with hematoxylin and eosin (H&E) as previously described. Lymphocytic infiltration was scored as described previously 24.

For immunofluorescence, the paraffin sections were deparaffinized, stained with primary antibodies (4°C, overnight), washed, incubated with secondary antibodies (room temperature, 2 h), and imaged with a Leica microscope. The following primary antibodies were used: anti-CD4 (Santa Cruz Biotechnology; Cat# sc-13573), anti-CD8 (Santa Cruz Biotechnology; Cat# sc-7970), anti-CD69 (Santa Cruz Biotechnology; Cat# sc-390889), and anti-CD103 (Santa Cruz Biotechnology; Cat# sc-376073).

### Confocal microscopy

T cells were plated on Cell-Tak (Corning)-precoated wells. For live-cell imaging, the cells were stained with MitoTracker Red (Beyotime; Cat# C1035), ER-Tracker Green (Beyotime; Cat# C1042), and Hoechst 33342 (Beyotime; Cat# C1022) to label the mitochondria, ER, and nuclei, respectively, and imaged on an Olympus FluoView FV10i.

For immunofluorescence, T cells were allowed to adhere, fixed, permeabilized, blocked, and stained with primary/secondary antibodies. Nuclei were counterstained with DAPI (Thermo Fisher Scientific, Waltham, MA, USA; Cat# D1306) and imaged with a Leica microscope. Detailed information concerning the primary antibodies is provided in Table S1.

### Transmission electron microscopy (TEM)

The samples were fixed (2.5% glutaraldehyde, 2 h, 4°C), postfixed (2% osmium tetroxide), dehydrated (graded ethanol, propylene oxide), and embedded (EMbed-812 resin). Ultrathin sections were stained (uranyl acetate and lead citrate) and observed on a PHILIPS CM120 transmission electron microscope.

### Seahorse extracellular flux assay

Metabolic flux was measured on a Seahorse XFe96 (Agilent Technologies, Santa Clara, CA, USA) as previously described 24. FAO effects were studied with 2.5 µM etomoxir. Exogenous and endogenous FA utilization was assessed using the XF Cell Mito Stress Test Kit, following a methodology similar to that described by Hsieh *et al*. 24.

### Measurement of ATP and MDA levels

Intracellular ATP (MedChemExpress, Monmouth Junction, NJ, USA; Cat# HY-K031) and MDA (Beyotime Biotechnology, Shanghai, China; Cat# S0131) levels were measured using kits according to the manufacturer's protocol, following an approach consistent with that of Li *et al*. 29.

### Liquid chromatography‒tandem mass spectrometry (LC‒MS/MS)

For targeted lipidomics, lipids were extracted (chloroform:methanol 1:2), and the lower organic layer was analyzed. The samples were detected on an ExionLCTM AD UPLC (Thermo Fisher Scientific, Waltham, MA, USA) coupled to a QTRAP® 6500^+^ tandem mass spectrometer. The data were processed with Analyst 1.6.3 (AB Sciex).

### PPARα transcriptional activity assay

PPARα transcriptional activity was evaluated using a dual-luciferase reporter assay. CD4^+^ T cells were cotransfected with a peroxisome proliferator response element (PPRE)-driven firefly luciferase reporter plasmid (Beyotime; Cat# D4285) and a Renilla luciferase plasmid as an internal control. Luciferase activity was measured using a dual-luciferase reporter assay system (Beyotime; Cat# RG088) 48 h after transfection and the indicated treatment.

### Statistical analysis

The data are presented as the means ± standard deviations (SDs). Statistical analysis was conducted with GraphPad Prism 10.0. *p* values were calculated via two-tailed unpaired Student's *t-*test (with Welch's correction if necessary) or ANOVA (Tukey's post hoc test). Significance was set at *p < 0.05.

## Results

### ACSL5 is upregulated upon T-cell activation and SS development

**Figure 1:**
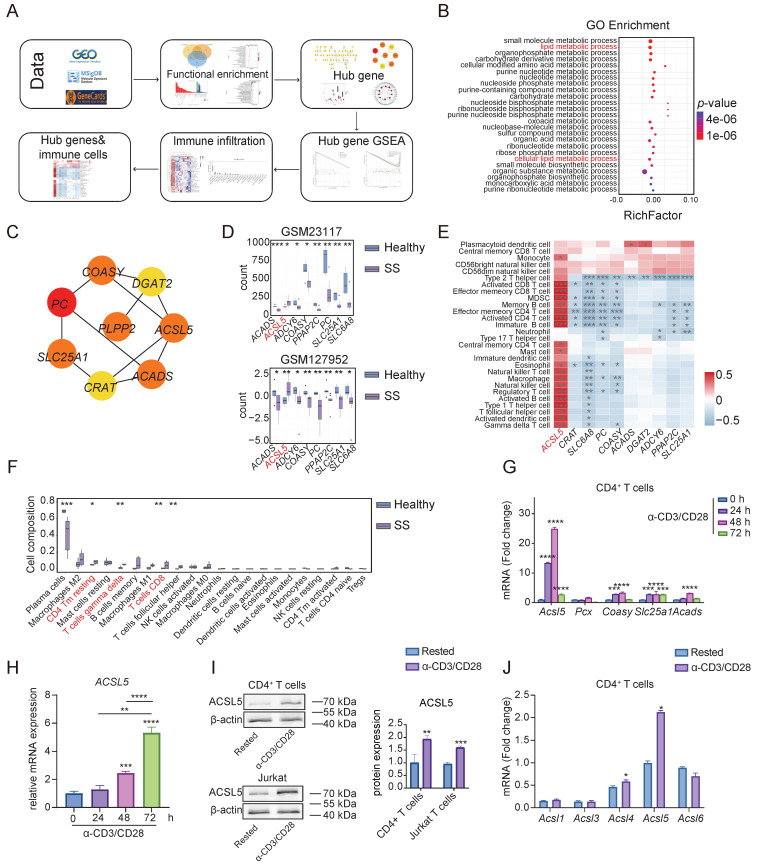
** ACSL5 is upregulated upon T-cell activation and SS development. (A)** Schematic illustration of RNA-seq screening workflow identifying ACSL5.** (B)** GO enrichment analysis of metabolism-related DEGs from patients in SS.** (C)** Hub genes identified via CytoHubba in SS patients.** (D)** Hub gene expression validation in datasets (GSM23117, GSM127952).** (E)** Spearman correlation between hub genes and immune cells in SS patients.** (F)** CIBERSORT analysis of immune cell fractions in the SS environment.** (G)** mRNA levels of hub genes in CD4^+^ T cells 72 h after stimulation with α-CD3/CD28.** (H)** ACSL5 mRNA levels in α-CD3/CD28-stimulated Jurkat T cells at 24, 48, and 72 h.** (I)** ACSL5 protein levels in rested and active CD4^+^ T and Jurkat T cells.** (J)** mRNA levels of *Acsl* isoforms in rested and active CD4^+^ T cells. The data are presented as the means ± SDs. n = 3 biological replicates for panels G-J. *p < 0.05, **p < 0.01, ***p < 0.001, ****p < 0.0001. Statistical analyses were performed using two-tailed tests, two-way ANOVA, and one-way ANOVA.

To explore potential metabolic biomarkers of SS development, we investigated publicly available RNA sequencing (RNA-seq) datasets (including GSE23117 and GSE127952) and compared differential gene expression between patients with SS and healthy controls (Figure 1A). A total of 70 differentially expressed metabolism-related genes (DEMRGs) were identified (Figure S1A-B). The enrichment analysis revealed that the DEMRGs were mostly associated with small-molecule catabolic processes and lipid metabolic processes according to the Gene Ontology (GO) terms (Figure 1B & Figure S1C). Kyoto Encyclopedia of Genes and Genomes (KEGG) and Reactome enrichment analyses also revealed a strong correlation between DEMRGs and lipid metabolism, including fatty acid metabolism and the peroxisome proliferator-activated receptor (PPAR) signaling pathway (Figure S1D-E), indicating that lipid metabolism may play a pivotal role in the pathogenesis of SS.

We next examined the interplay among these DEMRGs (Figure S1F). A total of 8 hub genes were identified within the PPI network (Figure 1C). Among these genes, *ACSL5* was consistently upregulated in infiltrating gland tissues (Figure 1D). Single-sample gene set enrichment analysis (ssGSEA) revealed that ACSL5 expression was strongly correlated with immune cell infiltration (Figure 1E). The CIBERSORT algorithm revealed elevated proportions of CD4 memory T cells, gamma delta (γδ) T cells, M1 macrophages, and CD8^+^ T cells infiltrating *in situ* in patients with SS (Figure 1F). Gene set enrichment analysis (GSEA) also revealed that *ACSL5* upregulation is related to autoimmune responses and cellular metabolic pathways (Figure S1G-H). To further confirm the role of ACSL5 in T-cell responses, primary CD4^+^ T cells and Jurkat T cells were activated by stimulation with anti-CD3 and anti-CD28 antibodies (α-CD3/CD28) (Figure S1I-J). ACSL5 was markedly upregulated in response to α-CD3/CD28 stimulation at the mRNA and protein levels (Figure 1G-I) and was prominently expressed in T cells among the ACSL family isoforms (Figure 1J). These findings suggest that ACSL5 is a key hub gene correlated with T-cell responses in SS.

### ACSL5 augments CD4^+^ T-cell activation and sustains a proinflammatory phenotype

**Figure 2:**
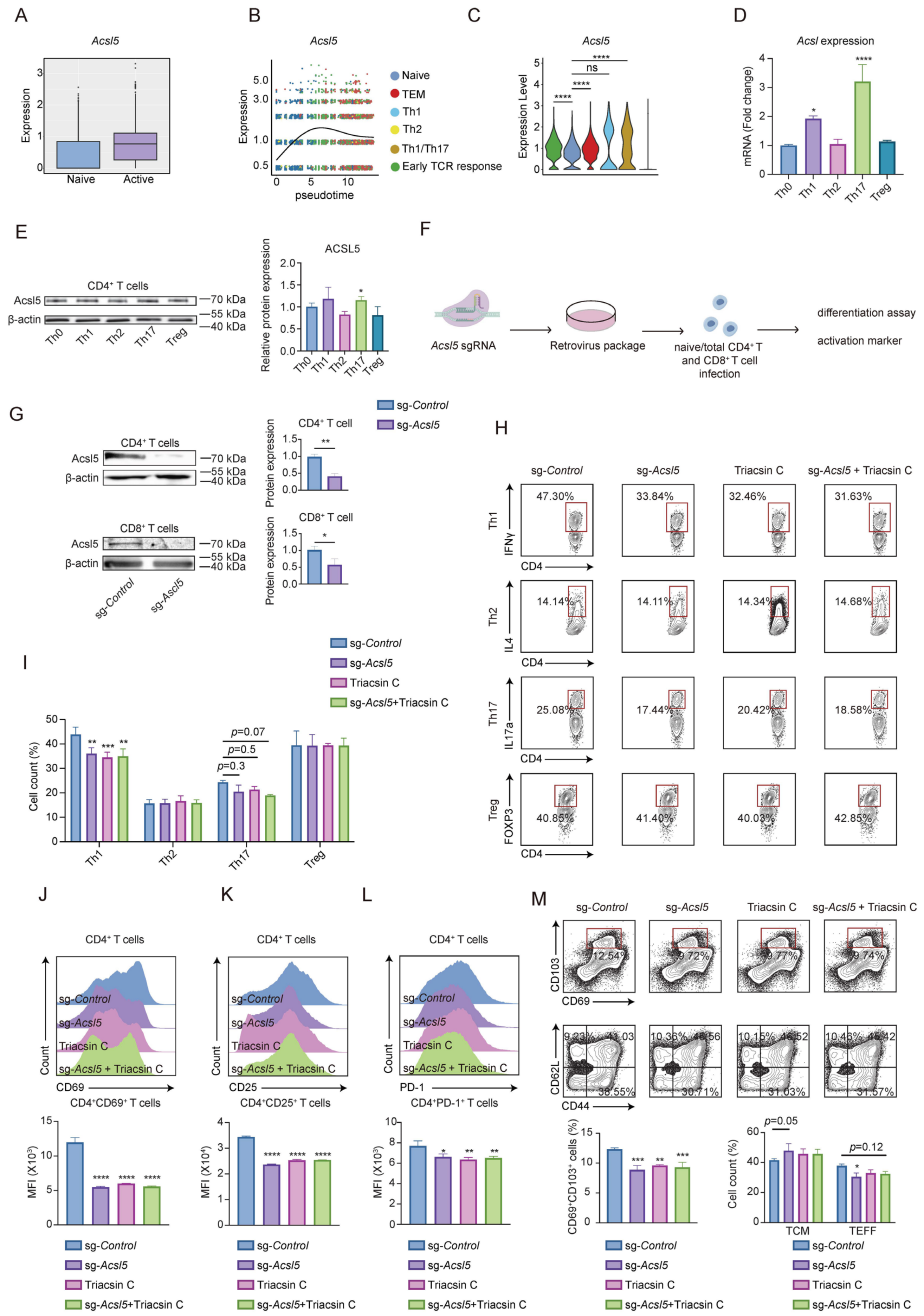
** ACSL5 augments CD4^+^ T-cell activation and sustains a proinflammatory phenotype. (A)** *Acsl5* expression in naïve and active CD4^+^ T cells according to the scRNA-seq data (GSE179623). **(B)** The mean *Acsl5* expression was plotted against the pseudotime values of the scRNA-seq data. **(C)** *Acsl5* gene expression in stimulated CD4^+^ T-cell subtypes according to the scRNA-seq data. **(D)** qPCR was used to measure the mRNA levels of *Acsl5* in CD4^+^ T-cell subtypes. **(E)** Protein expression of ACSL5 in CD4^+^ T-cell subtypes. **(F)** Schematic of *Acsl5* CRISPR/Cas9 knockout in primary T cells. **(G)** Validation of *Acsl5* knockout efficiency in CD4^+^ and CD8^+^ T cells. **(H)** Flow cytometric analysis of CD4^+^ T-cell differentiation after 6h of restimulation with PMA/ionomycin. **(I)** The percentages of IFNγ^+^, IL4^+^, IL17a^+^, and Foxp3^+^ T cells were quantified. **(J)** Flow cytometry of CD69 in CD4^+^ T cells at 72 h poststimulation. **(K)** Flow cytometric analysis of CD25 expression in CD4^+^ T cells at 72 h poststimulation. **(L)** Flow cytometry analysis of PD-1 expression in CD4^+^ T cells at 72 h poststimulation. **(M)** Flow cytometry of CD69^+^CD103^+^, CD44^+^CD62L^-^ and CD44^+^CD62L^+^ cells among CD4^+^ T cells at 72 h poststimulation. The data are presented as the means ± SDs. n = 3 biological replicates for panels D-M. *p < 0.05, **p < 0.01, ***p < 0.001, ****p < 0.0001. Statistical analyses were performed using two-tailed tests, two-way ANOVA and one-way ANOVA.

We next investigated the function of ACSL5 in T-cell responses. Analysis of the single-cell RNA sequencing (scRNA-seq) dataset (GSE179623) revealed that *Acsl5* expression was significantly upregulated and strongly correlated with T-cell activation (Figure 2A & Figure S2A-C). We then examined specific activation and differentiation phases and found that *Acsl5* expression progressively increased during activation and peaked following effector T-cell differentiation, particularly in the Th1 and Th17 cell subsets (Figure 2B & Figure S2D-F). We further examined ACSL5 expression across CD4⁺ T-cell subsets and found that ACSL5 was preferentially expressed in Th1 and Th17 cells, as confirmed by both the qPCR and WB results, whereas its expression remained relatively low in Th2 and Treg cells (Figure 2C-E).

We next used a retrovirus-packaged CRISPR/Cas9 single-guide RNA (sgRNA) to knock out *Ascl5* in primary T cells (Figure 2F). To comprehensively characterize the functional impact of ACSL5 on T-cell biology, we evaluated its role in T-cell activation and lineage-specific differentiation. The knockout efficiency in the indicated cells was validated (Figure 2G). *In vitro* differentiation assays of naïve CD4^+^ T cells into distinct effector T-cell subsets revealed that the proportions of interferon gamma (IFNγ)^+^CD4^+^ and interleukin 17A (IL17A)^+^CD4^+^ T cells were decreased after *Acsl5* knockout, whereas the proportions of interleukin-4 (IL4)+ T cells and Forkhead box P3 (FOXP3)+ T cells were largely maintained (Figure 2H-I). SgRNA-guided knockout of *Acsl5* in CD4^+^ T cells also decreased the expression of several activation markers, including CD69, CD25, and programmed cell death protein 1 (PD-1), whereas that in CD8^+^ T cells was relatively modest (Figure 2J-L & Figure S2G-I). The proportion of IFNγ^+^CD8^+^ T cells also decreased as a result of *Acsl5* deficiency, suggesting a compromised effector T-cell response (Figure S2J).

We further investigated its role in effector and memory T-cell transitions. ACSL5 deficiency significantly decreased the proportion of CD44^+^CD62L^-^ T cells within 72 h. Concurrent upregulation of the expression of CD69 and CD103 was detected, indicating increased tissue residency of T cells regulated by ACSL5 (Figure 2M). These findings suggest that ACSL5 may also be involved in the effector-to-memory transition of T cells.

We then explored a pharmacological approach to regulate the function of ACSL5. Given that ACSL5 is the predominant isoform expressed in activated T cells (Figure 1J & Figure S2K), we applied an inhibitor of ACSLs, namely, Triacsin C, to disrupt the function of ACSL5. Treatment with Triacsin C had a similar effect as treatment with *Acsl5* knockout did, and Triacsin C treatment failed to induce any additional phenotypic changes in *Acsl5*-knockout T cells, confirming the specificity of the drug for the ACSL5 pathway (Figure 2H-M & Figure S2G-I). Taken together, our results suggest that ACSL5 can promote CD4^+^ T-cell activation and sustain a proinflammatory signature.

### ACSL5 fuels fatty acid oxidation in CD4^+^ T cells through PPARα

**Figure 3:**
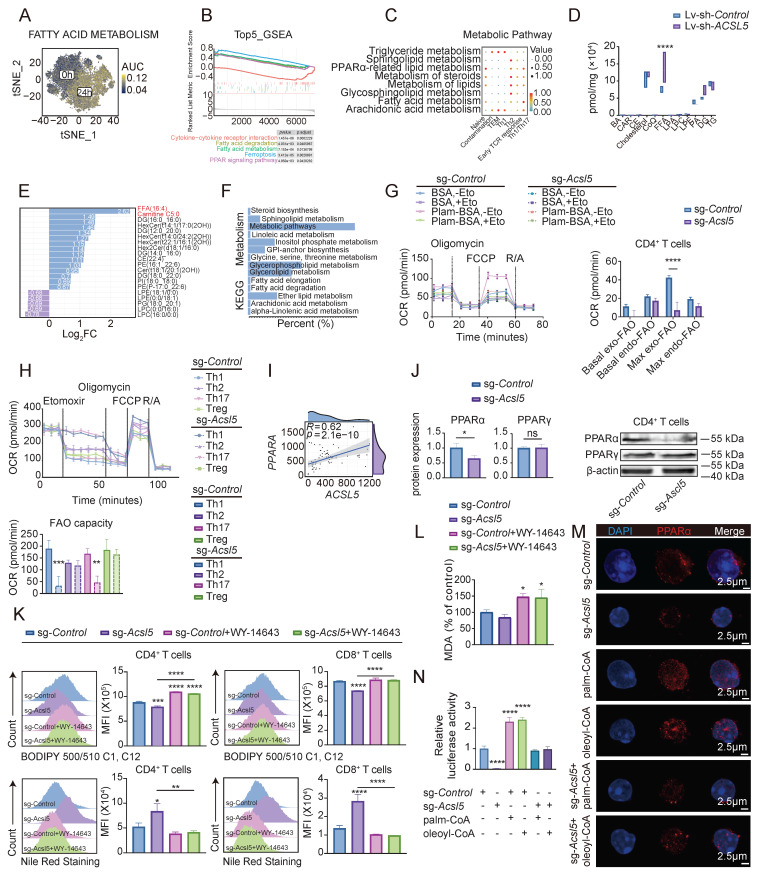
** ACSL5 fuels fatty acid oxidation in CD4^+^ T cells through PPARα. (A)** t-SNE plot reflecting fatty acid metabolism (AUCell). **(B)** GSEA of the top five pathways associated with ACSL5 expression. **(C)** ScMetabolism dot plot representing enriched metabolic pathways. **(D)** Box plot representing specific lipid abundances. **(E)** Bar plot of the top metabolites from lipid profiling. **(F)** KEGG enrichment analysis of upregulated lipids. **(G)** OCR analysis of CD4^+^ T cells treated with/without palmitate or etomoxir, with endogenous and exogenous FAO capacity quantified from the maximal OCR. **(H)** OCR analysis of the indicated CD4^+^ T-cell subsets (Th1, Th2, Th17 and Treg) transfected with sg-*Control* or sg-*Acsl5*, with FAO capacity quantified from etomoxir-sensitive respiration. **(I)** Correlation of *ACSL5* and *PPARA* expression in patients with SS. **(J)** PPARα and PPARγ protein levels in sg-*Control*- and sg-*Acsl5*-transfected CD4^+^ T cells. **(K)** Lipid uptake (BODIPY C12) and Nile red staining in sg-*Control*- and sg-*Acsl5*-transfected CD4^+^/CD8^+^ T cells.** (L)** MDA concentrations in sg-*Control*- and sg-*Acsl5*-transfected CD4^+^ T cells at 72 h poststimulation. **(M)** Confocal images of PPARα nuclear colocalization in sg-*Control*- and sg-*Acsl5*-transfected CD4^+^ T cells under the indicated oleoyl-CoA or palmitoyl-CoA treatment conditions. PPARα (red), nuclei (blue). Scale bar = 1 μm. **(N)** Dual-luciferase analysis of PPARα transcriptional activity in sg-*Control*- and sg-*Acsl5*-transfected CD4^+^ T cells under the indicated oleoyl-CoA or palmitoyl-CoA treatment conditions. The data are presented as the means ± SDs. n = 6 biological replicates for panels G-H, n = 82 biological replicates for panel I, and n = 3 biological replicates for panels D-F and J-N. *p < 0.05, **p < 0.01, ***p < 0.001, ****p < 0.0001. Statistical analyses were performed using two-tailed tests, two-way ANOVA, and one-way ANOVA.

We next aimed to elucidate the underlying mechanism of ACSL5 in CD4^+^ T cells. Previous studies have shown that ACSL5 is a pivotal enzyme of FAO and is deeply involved in T-cell fate 30. In line with previous research, increased fatty acid metabolism was identified in activated CD4^+^ T cells according to the scRNA-seq data of GSE179623 (Figure 3A). We then clustered and compared CD4^+^ T cells with *Acsl5*^high^, *Acsl5*^middle^, and *Acsl5*^low^ T cells and found that fatty acid metabolism and acyl-CoA biosynthetic processes were strongly correlated with cytokine interactions and the PPAR signaling pathway in *Acsl5*^high^ CD4^+^ T cells (Figure 3B & Figure S3A-B). In fact, both CD4^+^ T cells with early T-cell receptor (TCR) responses and Th1/Th17 differentiation require elevated fatty acid metabolism, including sphingolipid metabolism, peroxisome proliferator-activated receptor alpha (PPARα) signaling, and steroid metabolism (Figure 3C).

We also applied a lentivirus containing short hairpin RNA (shRNA) against *ACSL5* (sh-*ACSL5*) to Jurkat T cells to investigate the long-term T-cell response (Figure S3C-D). We applied LC-MS analysis to detect specific metabolic changes upon *ACSL5* depletion*.* The results revealed a markedly increased abundance of FAs and carnitine in *ACSL5*-knockdown Jurkat T cells (Figure 3D). Interestingly, *ACSL5* knockdown led to increased concentrations of most first-class lipid categories, except for lysophosphatidylcholine (LPC), lysophosphatidylethanolamine (LPE), phosphatidic acid (PA), and phosphatidylcholines (PCs), suggesting decreased lipid turnover (Figure 3E). These different metabolites were mostly related to fatty acid metabolism (Figure 3F). The intracellular FA pool depends on intracellular synthesis and exogenous uptake 31. Furthermore, FA uptake was decreased in *Acsl5*-knockout CD4^+^ T cells (Figure S3E). We then detected the mitochondrial FAO capacity in response to internal fatty acid and exogenous fatty acid supplementation. The maximal oxygen consumption rate (OCR) of *Acsl5*-knockout primary T cells revealed marked exogenous FAO impairment (Figure 3G). Furthermore, etomoxir treatment of Jurkat T cells *in vitro* reduced T-cell activation and proliferation (Figure S3F). Given its preferential expression and effects on proinflammatory T cells, we next investigated whether ACSL5-FAO was selective for distinct CD4^+^ T-cell subsets. We found that *Acsl5* knockout led to a pronounced reduction in the FAO capacity of Th1 and Th17 cells, whereas that of Treg and Th2 cells remained largely unchanged. Taken together, these findings indicate that proinflammatory CD4^+^ T-cell subsets are more dependent on ACSL5-driven FAO to support their metabolic demands (Figure 3H).

Through correlation analysis of the transcriptional profiles of patients with SS, we found that *ACSL5* expression is strongly correlated with the expression of the lipid metabolism molecule PPARα in CD4^+^ T cells during the development of SS (Figure 3I). Western blot analysis also revealed that ACSL5 deficiency downregulated PPARα expression at the protein level (Figure 3J-Figure S3G-H). Therefore, we investigated whether ACSL5 regulates FAO through PPARα. To validate this, we pharmacologically reactivated PPARα signaling with the selective agonist WY-14643 and assessed the global lipid metabolic state. WY-14643 treatment restored fatty acid uptake and MDA levels in *Ascl5*-knockout T cells (Figure 3K-L). Given that triglycerides (TGs) stored in lipid droplets (LDs) can be mobilized to generate free fatty acids (FFAs) that fuel β-oxidation 32, we next evaluated LD dynamics. *Acsl5* deficiency in CD4^+^ T cells led to marked LD accumulation, whereas WY-14643 significantly reduced LD size and abundance (Figure 3K). Consistent with these findings, lipidomic analysis revealed lipid overload upon *ACSL5* knockdown in Jurkat T cells (Figure S3I). These results suggest that ACSL5/PPARα improves the lipolysis of TGs stored in LDs and provides FAs for FAO in T cells. We discovered that nuclear PPARα abundance was reduced after *Acsl5* knockout, which was accompanied by decreased PPARα transcriptional activity (Figure S3J-K). Given that ACSL5 is localized to the endoplasmic reticulum and outer mitochondrial membrane, we investigated whether ACSL5-derived acyl-CoA species serve as functional ligands for PPARα. Supplementation with fatty acyl-CoA species, including palmitoyl-CoA and oleoyl-CoA, which are representative intermediates preferentially generated by ACSL5, increased PPARα nuclear localization and transcriptional activity, as demonstrated by confocal microscopy and dual-luciferase reporter assays (Figure 3M-N), indicating that ACSL5 promotes the generation of activated fatty acid intermediates, thereby facilitating ligand-dependent activation of PPARα. In summary, we proposed that ACSL5 supports FAO through PPARα to support T-cell responses.

### The ACSL5-PPARα axis regulates MFN2-related mitochondrial function to influence FAO

**Figure 4:**
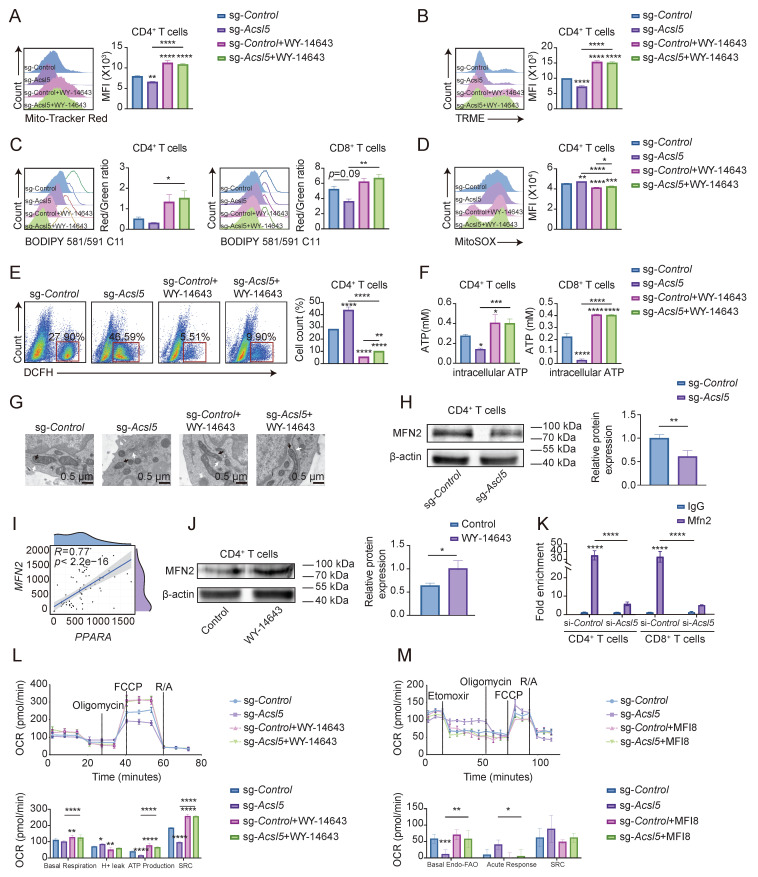
** The ACSL5-PPARα axis regulates MFN2-related mitochondrial function to influence FAO. (A)** Representative flow images of MitoTracker Red staining, which was used to assess mitochondrial mass in sg-*Control*- and sg-*Acsl5*-transfected CD4^+^ T cells ± WY-14643. **(B)** Quantification of the mean TMRE fluorescence intensity (MFI), which represents the mitochondrial membrane potential, in sg-*Control*- and sg-*Acsl5*-transfected CD4^+^ T cells with/without WY-14643. **(C)** Lipid peroxidation (BODIPY C11) in sg-*Control*- and sg-*Acsl5*-transfected CD4^+^/CD8^+^ T cells. **(D)** MitoSOX Red MFI in sg-*Control*- and sg-*Acsl5*-transfected CD4^+^ T cells ± WY-14643. **(E)** Intracellular ROS levels were assessed via flow cytometry. **(F)** Luciferase-based ATP assay in CD4^+^/CD8^+^ T cells 72 h poststimulation. **(G)** TEM of the mitochondrial structure and MERCs of sg-*Control*- and sg-*Acsl5*-transfected CD4^+^ T cells with/without WY-14643. Arrows: black (mitochondria), white (ER). Scale bar = 0.5 μm. **(H)** Immunoblotting of MFN2 in sg-*Control*- and sg-*Acsl5*-transfected CD4^+^ T cells. **(I)** Correlation analysis of PPARA and MFN2. **(J)** Immunoblotting analysis showing MFN2 expression in the WY-14643-treated and control CD4^+^ T cells after 24h. **(K)** ChIP‒qPCR analysis of PPARα at the MFN2 promoter in CD4^+^ and CD8^+^ T cells. **(L)** Mitochondrial stress test (OCR) of the indicated groups of CD4^+^ T cells. **(M)** Effects of mitochondrial and FAO inhibitors on the OCR in CD4^+^ T cells. The data are presented as the means ± SDs; n = 6 biological replicates for panels L-M; n = 82 cells for panel I; and n = 3 biological replicates for panels A-H. *p < 0.05, **p < 0.01, ***p < 0.001, ****p < 0.0001. Statistical analyses were performed using two-tailed tests, two-way ANOVA and one-way ANOVA.

Since the metabolic flux of FAO is fundamentally linked to mitochondrial function in T cells 33, we investigated whether mitochondria are involved in the regulatory loop of the ACSL5/PPARα axis. *Acsl5* knockout in primary CD4^+^ T cells led to a reduction in mitochondrial mass, as indicated by MitoTracker Red staining, and a decrease in mitochondrial membrane potential (MMP), as measured by TMRE fluorescence intensity (Figure 4A-B), accompanied by increased lipid peroxidation, mitochondrial superoxide, and reactive oxygen species (ROS) production, indicating increased oxidative stress (Figure 4C-E). This effect was reversed upon WY-14643 administration (Figure 4C-E). Similarly, compared with Triacsin C treatment, long-term depletion of *ACSL5* in Jurkat T cells and Triacsin C treatment resulted in decreased mitochondrial mass and elevated oxidative stress (Figure S4A-E). Furthermore, the inhibition of FAO resulted in the scavenging of ROS to improve mitochondrial function (Figure S4F). Mitochondrial dysfunction led to decreased ATP synthesis in both *Acsl5*-deficient T cells and *ACSL5*-knockdown Jurkat T cells (Figure 4F & Figure S4G). These results show that the ACSL5-PPARα axis altered mitochondrial function in T cells.

We then investigated how ACSL5-regulated FAO flux affects mitochondrial biology. Since alterations in mitochondrial dynamics can affect the mass and quality of mitochondria 34, we observed mitochondrial morphology via TEM, which revealed that the control group and the WY-14643-rescue group exhibited elongated mitochondria, increased cristae density, and a higher frequency of mitochondrial-ER contacts (MERCs) (Figure 4G & Figure S4H). Furthermore, the expression of the pivotal mitochondrial dynamic protein mitofusin 2 (MFN2) decreased in the absence of ACSL5, whereas the expression of other mitochondrial dynamic proteins, including optic atrophy 1 (OPA1) and mitofusin 1 (MFN1), was comparable (Figure 4H & Figure S4I). MFN2 plays a key role in the fusion of mitochondria, tethering mitochondria to the endoplasmic reticulum (ER), and indicating metabolic changes 35. Bioinformatics analysis revealed a strong positive correlation between *PPARA* and *MFN2* in patients with SS (Figure 4I). Additionally, pharmacological activation of PPARα with WY-14643 increased MFN2 expression, as detected by immunoblotting (Figure 4J). Since PPARα is a widely acknowledged transcription factor that modulates the transcription of downstream genes, including MFN2 36, we therefore proposed that the ACSL5/PPARα axis regulates mitochondrial shift through increased *Mfn2* transcription activity. The direct binding of PPARα to the *Mfn2* promoter was confirmed via a ChIP assay in CD4^+^ and CD8^+^ T cells and was shown to be regulated by *Ascl5* (Figure 4K), suggesting that ACSL5 facilitated nuclear translocation of PPARα, thereby increasing *Mfn2* transcription in T cells.

We next investigated changes in MFN2-related mitochondrial function. Confocal microscopy revealed more fragmented mitochondria and a lower frequency of MERCs in *Acsl5*-knockdown CD4^+^ and CD8^+^ T cells, whereas WY-14643 treatment partially reversed these effects, as verified by the knockdown efficiency (Figure S4J-K). MERCs play pivotal roles in multiple lipid metabolism pathways and are indispensable for lipid exchange 37. Lipidomic analysis further supported the increase in membrane lipid exchange (Figure S4L). Confocal microscopy demonstrated that* Mfn2* knockdown markedly reduced the number of MERCs, and these effects were confirmed by WB, whereas supplementation with palmitoyl-CoA and ACSL5-derived lipid intermediates partially reversed these effects (Figure S4M-N). Together, these findings revealed that ACSL5/PPARα regulated mitochondrial function, improving the ability of MFN2 to enable mitochondrial fusion and the mitochondria-ER connection. *Mfn2* knockdown significantly impaired FAO capacity, whereas palmitoyl-CoA supplementation reversed this effect, indicating that ACSL5/MFN2-dependent MERCs are functionally required for efficient FAO (Figure S4O).

FAO-dependent T cells are characterized by enhanced spare respiratory capacity (SRC) 38. The seahorse results confirmed a lower OCR and SRC in *Acsl5*-knockout CD4^+^ T cells, whereas the addition of WY-14643 increased the SRC, indicating possible compensation by PPARα in the absence of ACSL5 (Figure 4L). To determine whether the ACSL5/PPARα-induced increase in mitochondrial SRC was attributed to MFN2, we performed a Seahorse Mito stress test, which included acute injection of etomoxir. Nearly all respiratory parameters decreased markedly in the control group following etomoxir injection, whereas the inhibitory effect of MF18, a well-established inhibitor of MFN2, was reversed. Conversely, etomoxir did not affect mitochondrial function in *Acsl5*-knockout cells (Figure 4M).

### ACSL5/PPARα/FAO-mediated mitochondrial function determines T-cell fate

**Figure 5:**
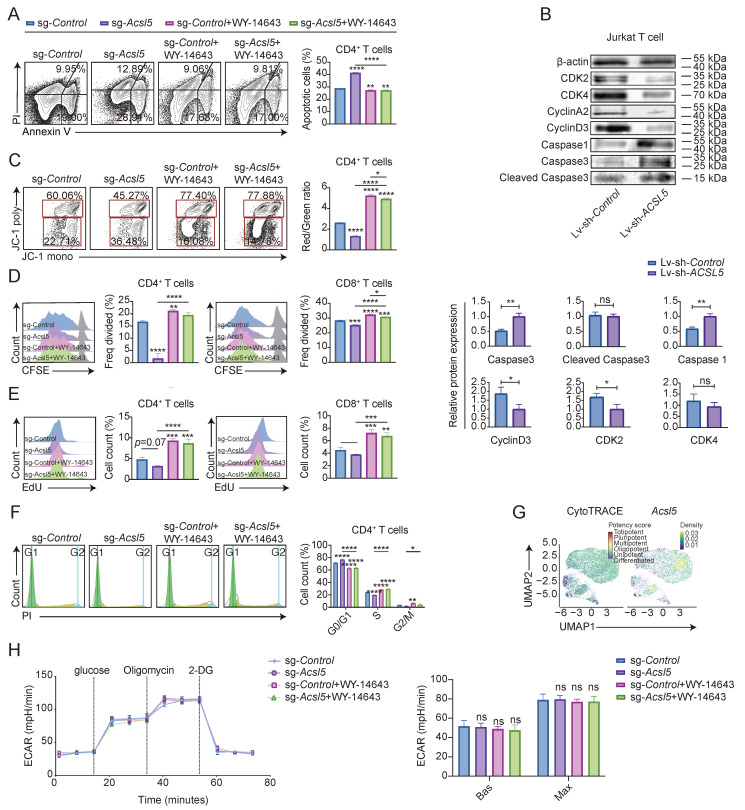
** ACSL5/PPARα/FAO-mediated mitochondrial function determines T-cell fate. (A)** Flow cytometry analysis of CD4^+^ T-apoptosis. **(B)** Western blot analysis of cell cycle (CDK2/4 and cyclin A2/D3) and apoptosis (caspase-1/3) proteins in Lv-sh-*Control* vs. Lv-sh-*ACSL5* Jurkat T cells. **(C)** Detection of JC-1 fluorescence in the indicated cells via flow cytometry. **(D)** CFSE proliferation assay of CD4^+^ and CD8^+^ T cells at 72 h poststimulation. **(E)** EdU proliferation assay of CD4^+^ and CD8^+^ T cells at 72 h poststimulation. **(F)** Cell cycle analysis of CD4^+^ T cells by flow cytometry at 72 h poststimulation. **(G)** UMAP plots of CytoTRACE scores and stemness in stimulated CD4^+^ T cells. **(H)** Glycolytic stress test and glycolytic capacity of sg-*Control*- and sg-*Acsl5*-transfected CD4^+^ T cells with/without WY-14643. The data are presented as the means ± SDs. n = 6 biological replicates for panel H, and n = 3 biological replicates for panels A-F. *p < 0.05, **p < 0.01, ***p < 0.001, ****p < 0.0001. Statistical analyses were performed using two-tailed tests, two-way ANOVA and one-way ANOVA.

We next aimed to determine the effect of ACSL5/PPARα/FAO on the T-cell phenotype. Given that elevated mitochondrial FAO promotes T-cell longevity 39 and a memory phenotype 40, 41, we further examined T-cell phenotypes beyond differentiation regulated by the ACSL5/PPARα/FAO axis. JC-1 and Annexin V/PI probes increased the apoptotic ratios of *Acsl5*-deficient CD4^+^ and CD8^+^ T cells. A similar trend was also observed in Jurkat T cells with stable knockdown of ACSL5 (Figure 5A-C & Figure S5A-B). A decrease in proliferative capacity was also observed according to the results of carboxyfluorescein succinimidyl ester (CFSE), 5-ethynyl-2'-deoxyuridine (EdU), and Ki67 staining (Figure 5D-E & Figure S5C-D). Additionally, *ACSL5* deficiency or knockdown was associated with inhibition of the S phase of the cell cycle (Figure 5F & Figure S5E). Notably, WY-14643 treatment completely restored the apoptotic and proliferative activity of *Acsl5*-knockout T cells and* ACSL5*-knockdown Jurkat T cells (Figure 5A-F). Cluster analysis of the scRNA-seq data revealed that *ACSL5* was positively associated with T-cell differentiation but preserved a certain degree of stemness before terminal differentiation (Figure 5G). These findings demonstrated that ACSL5 inhibited terminal differentiation and apoptosis while promoting T-cell proliferation. To understand the metabolic basis for this increase in proliferation and survival, we next investigated whether ACSL5 influenced key metabolic pathways. We discovered that glycolytic activity remained stable in *Acsl5*-knockout T cells (Figure 5H), suggesting that ACSL5 has little secondary effect on glycolysis. Collectively, these data indicate that ACSL5/PPARα/FAO endows T cells with increased metabolic fitness, thereby enhancing their survival capacity.

**Figure 6:**
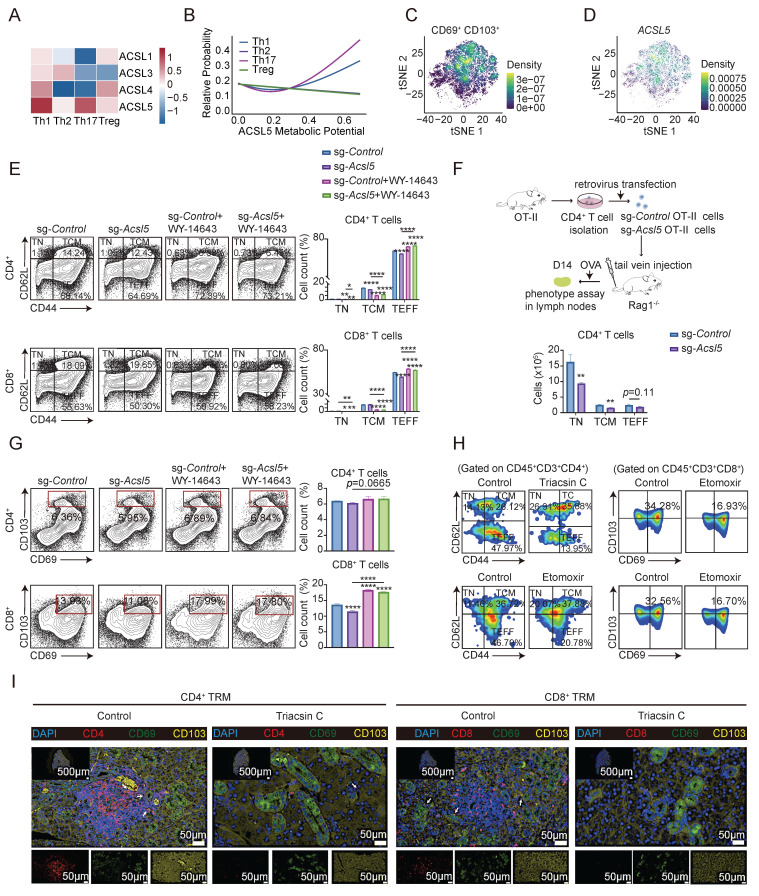
** ACSL5/PPARα/FAO-mediated mitochondrial function determines T-cell fate. (A)** Expression of *ACSL* family members in T-cell subsets in labial salivary gland tissues from adults with Sjögren's syndrome and controls. **(B)** Differentiation probabilities of T-cell subsets according to *ACSL5* expression. **(C)** Feature plot of the TRM markers *CD69* and *ITGAE (CD103)* in T cells. **(D)** UMAP plot showing *ACSL5* expression. **(E)** Representative CD44/CD62L flow plots and quantification of T-cell subsets (TCMs and TEFFs) among CD4^+^/CD8^+^ T cells. **(F)** Schematic of OT-II CD4^+^ T-cell adoptive transfer and *in vivo* differentiation analysis 14 days after T-cell transfer. **(G)** Flow cytometry of CD69^+^CD103^+^ cells among CD4^+^ and CD8^+^ T cells at 72 h poststimulation. **(H)** T-cell memory (CD44^+^CD62L^+^) and TRM (CD69^+^CD103^+^) subsets in the submandibular glands (SMGs) of FAO inhibitor-treated and saline-treated NOD mice at 12 weeks of age. **(I)** Immunofluorescence staining for CD4/8, CD69, and CD103 in SMG tissue from 12-week-old SS-like mice. Arrows: colocalization. Nuclei (nuclei). Scale bar = 50-500 μm. The data are presented as the means ± SDs. n = 5 biological replicates for panels H-I, and n = 3 biological replicates for panels E-G. *p < 0.05, **p < 0.01, ***p < 0.001, ****p < 0.0001. Statistical analyses were performed using two-tailed tests, two-way ANOVA, and one-way ANOVA.

We also investigated the role of the ACSL5/PPARα/FAO axis in global T-cell fate in addition to terminal differentiation. In the public scRNA dataset from the salivary glands of SS patients (GSE272409), we identified 8 distinct clusters and then subdivided the T cells into 11 subsets (Figure S6A-C). Given the selective effects of ACSL5 on CD4⁺ T-cell differentiation described above, we next investigated whether ACSL5 exhibits functional associations across T-cell lineages. The results revealed that ACSL5 was preferentially enriched in Th1 and Th17 cells, whereas the Treg and Th2 subsets appeared to rely more strongly on other ACSL isoforms (Figure 6A). Consistently, ACSL5 expression was positively associated with Th1/Th17 transcriptional programs but weakly associated with FOXP3-driven Treg programs (Figure S6D). Lineage probability models of ACSL5 expression revealed that increasing ACSL5 expression was associated with a progressive increase in Th1 and Th17 lineage probabilities, whereas that in Tregs remained largely unchanged (Figure 6B). Taken together, these results demonstrate that ACSL5 preferentially supports Th1/Th17 differentiation programs but is limited in its involvement in Treg lineage commitment.

Cell composition analysis revealed a general increase in tissue-resident memory T (TRM) cells in SS patients with active fatty acid metabolism (Figure S6E-F). Strikingly, high *ACSL5* expression was correlated with TRM markers, suggesting a potential role in TRM formation (Figure 6C-D). We then explored the effect of the ACSL5/PPARα/FAO axis on the effector-memory sustainability of T cells. *Acsl5* knockout mainly reduced the proportions of CD4^+^ and CD8^+^ effector T (TEFF) cells (CD44^+^ CD62L^-^), whereas central memory-like T (CD44^+^ CD62L^+^) cells were moderately affected. TEFF expansion was observed following WY-14643 treatment, which is consistent with previous findings (Figure 6E). In addition, *in vitro* incubation with Triacsin C resulted in fewer TEFF-like cells, whereas the number of central memory T (TCM)-like cells moderately decreased (Figure S6G).

To elucidate the cell-intrinsic role of ACSL5 in T-cell fate determination *in vivo*, we applied adoptive transfer of T cells isolated from OT-II transgenic mice into Rag1^-^/^-^ recipient mice, followed by sgRNA-guided *Acsl5* knockout. The results revealed decreased populations of TEFF and TCM in the lymph nodes (Figure 6F & Figure S6H). Our next quest for the role of ACSL5 extends to other T-cell differentiation programs, particularly the formation of TRMs, as indicated previously. We observed a significant decrease in CD8^+^CD69^+^CD103^+^ T cells and a comparable downward trend in the CD4^+^ compartment. Notably, the administration of WY-14643 restored the TRM phenotype in both subsets (Figure 6G). To determine its effect *in vivo*, we treated SS-like model mice with etomoxir or Triacsin C. The proportions of TCM, TEFF and TRM cells revealed that ACSL5/PPARα/FAO promotes the formation and maintenance of memory T cells (Figure 6H & Figure S6I). The immunofluorescence results confirmed that the inhibition of ACSL5 or FAO considerably prevented local TRM formation (Figure 6I & Figure S6J). Consistent with these findings, palmitoyl-CoA supplementation partially restored the decreases in Th1/Th17 ratios and attenuated effector function in *Acsl5*-deficient cells, an effect that was subsequently abrogated upon MFN2 inhibition (Figure S6K). Taken together, these data suggest that FAO sustained by the ACSL5/PPARα/MFN2 axis selectively supports the metabolic demands of inflammatory T-cell responses in the SjS microenvironment.

### Targeting ACSL5 and FAO reverses SS symptoms *in vivo*

**Figure 7:**
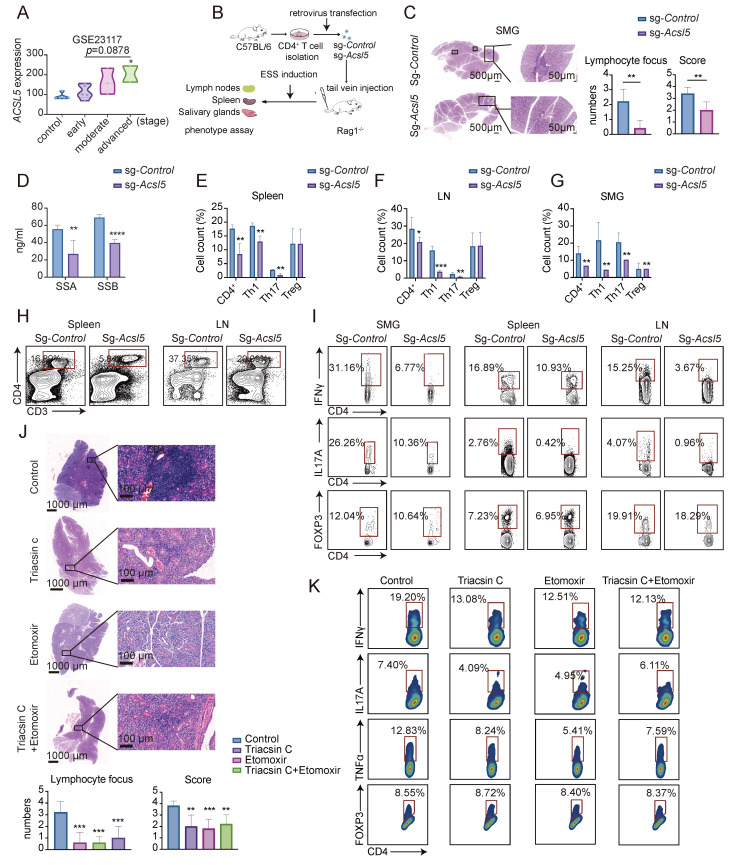
** Targeting ACSL5 and FAO reverses SS symptoms *in vivo*. (A)** Correlations of *ACSL5* expression with disease stage (GSE23117). **(B)** Schematic of the adoptive transfer of CD4^+^ T cells, followed by ESS induction and *in vivo* evaluation of disease severity and T-cell phenotypes. **(C)** H&E staining of representative salivary glands and quantification of histological scores and lymphocyte foci (scale bar = 50-500 μm). The black boxes indicate lymphocytic foci. **(D)** Serum SSA and SSB levels in the indicated groups. **(E)** CD4^+^ T-cell and T-cell subpopulation ratios in the spleens of the indicated groups. **(F)** CD4^+^ T-cell and T-cell subpopulation ratios in the lymph nodes of the indicated groups. **(G)** CD4^+^ T-cell and T-cell subpopulation ratios in the submandibular salivary glands of the indicated groups. **(H)** Representative plots of CD4^+^ T cells in the spleen and lymph nodes of the indicated groups.** (I)** Representative plots of T-cell subpopulations in the spleen, lymph nodes, and submandibular salivary glands of the indicated groups.** (J)** H&E staining of representative salivary glands and quantification of disease severity (scale bar = 100-1000 μm). **(K)** Flow cytometry of T-cell subpopulations in Triacsin C/etomoxir-treated vs. control mice at 12 weeks of age. The data are presented as the means ± SDs. n = 15 biological replicates for panel A, and n = 5 biological replicates for panels C-K. *p < 0.05, **p < 0.01, ***p < 0.001, ****p < 0.0001. Statistical analyses were performed using two-tailed tests, two-way ANOVA, and one-way ANOVA.

Finally, we investigated whether targeting the ACSL5/FAO axis could prevent SS progression *in vivo. ACSL5* expression was positively correlated with lymphocytic infiltration, disease stage, and systemic disease activity (Figure 7A & Figure S7A-E). To evaluate the *in vivo* role of ACSL5, we performed adoptive transfer of sg-*Acsl5*-modified CD4⁺ T cells followed by ESS induction (Figure 7B). Histological analysis of the salivary glands revealed significantly reduced inflammatory infiltration, as evidenced by lower histological scores and fewer lymphocytic foci (Figure 7C). Consistently, serum levels of autoantibodies, including those of SSA and SSB, were markedly reduced (Figure 7D). In contrast, the TGF-β1 levels remained comparable (Figure S7F). Flow cytometric results further revealed a significant reduction in CD4⁺ T-cell infiltration in the salivary glands, lymph nodes, and spleen (Figure 7E-H). Subset analysis demonstrated that Th1 and Th17 proportions markedly decreased across these tissues, whereas Treg and Th2 populations remained largely unaffected (Figure 7I). These findings indicate that ACSL5 functions within T cells and preferentially promotes the response of pathogenic T-cell subsets *in vivo*.

To further validate these findings, pharmacological inhibition using Triacsin C or etomoxir led to comparable results. Treatment significantly reduced serum IL-17 and IFN-γ levels and alleviated salivary gland inflammation, as reflected by lower histological scores and decreased lymphocytic infiltration (Figure 7J & Figure S7G). Reduced numbers of CD4⁺ and CD8⁺ T cells and a marked downregulation of proinflammatory cytokines at the mRNA level were observed, whereas the expression of Tgfb1 changed only modestly, with no significant difference at the protein level (Figure S7H-K). Consistently, intracellular staining revealed decreased proportions of IFNγ-producing, IL-17A-producing, and tumor necrosis factor alpha (TNFα)-producing CD4^+^ T cells, suggesting suppressed effector differentiation, while the proportion of Tregs remained comparable (Figure 7K & Figure S7L). Collectively, these results indicate that pharmacological inhibition of ACSL5/FAO can effectively treat SS-like symptoms *in vivo*.

## Discussion

Our study provides a previously unexplored immunometabolic perspective on the mechanistic role of ACSL5. ACSL5 has traditionally been characterized primarily as an enzyme that catalyzes the conversion of long-chain fatty acids (LCFAs) to LCFA-CoA for β-oxidation 42. ACSL5 is localized to the outer mitochondrial membrane and ER and is known to facilitate lipid oxidation 43, with implications in cancer and metabolic disorders 44, 45. While elevated ACSL5 transcript levels have been reported in systemic lupus erythematosus (SLE) patients 46, its mechanistic role in T-cell-mediated autoimmunity remained largely unexplored before this investigation. Here, we demonstrate that ACSL5 acts as a critical metabolic checkpoint that governs mitochondrial fitness via a noncanonical PPARα-MFN2 axis, thereby sustaining the pathogenicity and persistence of infiltrating inflammatory T cells in SS.

Targeting immune cell metabolism is promising for clinical intervention 47. Glucose 48, fatty acid 49, and amino acid metabolism are key regulators of T-cell fate, modulating immunity via energy supply, signal transduction, epigenetic regulation, and organelle biogenesis 50. Specifically, FAO plays an intricate and enabling role during the initial phases of T-cell activation and is required for the switch to catabolic pathways that sustain long-term effector function 51,52. Although FAO is often linked to Treg biology, our data indicate that ACSL5-dependent metabolic reprogramming preferentially supports pathogenic effector and memory T-cell persistence but has relatively limited effects on the Treg compartment. These findings suggest that in the nutrient-restricted microenvironment of the inflamed exocrine gland, FAO serves not as a lineage-specific signal for suppression but as a survival requirement for effector persistence 53. Consistent with this, emerging evidence indicates that CPT1A-dependent long-chain FAO is not strictly required for Treg differentiation, as genetic ablation of CPT1A does not impair Treg development 54. Our findings also support that ACSL5-dependent lipid metabolism is preferentially engaged in inflammatory CD4^+^ T-cell subsets, particularly Th1 and Th17 cells, which exhibit increased ACSL5 expression in SS. Since different ACSL family members preferentially generate different lipid intermediates, ACSL5-derived intermediates may preferentially sustain Th1/Th17-associated metabolic programs, increasing their sensitivity to ACSL5 inhibition. Accordingly, inhibition of ACSL5-mediated FAO disproportionately affects these subsets, whereas compared with control cells, Treg cells and Th2 cells, which have lower ACSL5 expression and alternative metabolic reliance, these cells are relatively insensitive. Notably, efficient FAO can also mitigate intracellular lipid overload by channeling fatty acids away from potentially harmful intermediates, thereby reducing metabolic stress. In this context, ACSL5 may provide inflammatory T cells with the metabolic capacity to resist exhaustion and maintain a long-lived, tissue-adapted phenotype. Furthermore, in addition to metabolic enzymes, metabolites themselves—such as fatty acids—act as signaling mediators 55. Manzo, T *et al.* recently reported that ACSL5 substrates (e.g., palmitic and oleic acid) profoundly impact T-cell reprogramming 56, suggesting that ACSL5-dependent lipid species may contribute to downstream signaling programs. Whether specific fatty acids act cooperatively with ACSL5-driven pathways will be an important direction for future work.

Mechanistically, we revealed that ACSL5 functions beyond its classical enzymatic role, triggering mitochondrial dynamics via a noncanonical ACSL5-PPARα-MFN2 axis. Interactions between FAO and mitochondrial dynamics have been reported; for example, Mouton, F. *et al*. observed their crosstalk 57. Our study further clarified the specific molecular signal transduction pathways involved. In addition to FAO, mitochondrial dynamics and cristae remodeling are critical determinants of T-cell immune responses 58. Specifically, mitochondrial fusion, mediated by proteins such as MFN1 and MFN2, protects mitochondria from stress and is vital for robust energy production 59. MFN2 also affects mitochondria-ER contact sites, which serve as hubs for efficient lipid exchange and transport into mitochondria for β-oxidation 60. Our study revealed that the ACSL5/PPARα/MFN2 pathway governs mitochondrial dynamics and endoplasmic reticulum crosstalk in inflammatory T cells. Crucially, the remodeling of mito-ER contacts acts as a key intermediate coupling ACSL5-mediated lipid metabolism to mitochondrial fatty acid oxidation, thereby actively modulating inflammatory T-cell responses 61. Collectively, our findings, in addition to those of previous reports, highlight the pivotal role of the mitochondria-centric orchestration of T-cell immunity in autoimmune pathology.

To directly investigate the role of ACSL5 in antigen-driven T-cell responses* in vivo*, we employed an adoptive transfer system using OT-II-derived CD4^+^ T cells. This approach enables a synchronized response to OVA stimulation and allows a more controlled assessment of *Acsl5*-dependent T-cell behavior 62. In parallel, we also used conventional C57BL/6 CD4^+^ T-cell transfer followed by ESS induction as a complementary disease-relevant validation model. Consistent with our *in vitro* findings, the transfer of *Acsl5*-deficient T cells selectively reduced effector Th1/Th17 responses and attenuated local inflammation. Our study revealed that ACSL5 primarily affects effector T-cell persistence and memory T-cell survival, both of which are critical drivers of SS pathogenesis. Effector T cells contribute to direct glandular damage and inflammation 63, whereas memory T cells maintain the autoimmune reservoir, leading to disease chronicity and relapse 64. Consequently, targeting ACSL5 represents a promising therapeutic strategy for autoimmune therapy.

However, several limitations should be noted. Pharmacological inhibitors, such as Triacsin C and etomoxir, lack strict target specificity. Therefore, etomoxir was used in the present study as a selective pharmacological approach to inhibit the FAO process. ACSL5 is widely expressed in both lymphocytes and epithelial cells 65. Although the adoptive transfer approach minimizes potential confounding effects from off-target activity or effects not specific to T cells, future studies employing more specific models will be needed to further validate these findings. Furthermore, whether ACSL5 modulates other immune cell lineages or whether PPARα recruits additional cofactors remains to be determined. Future investigations will focus on elucidating the upstream mechanisms responsible for the specific upregulation of ACSL5 in SS, as well as the discovery and development of targeted inhibitors against the ACSL5-fatty acid metabolic axis.

Collectively, our findings identify ACSL5 as a metabolic checkpoint that sustains pathogenic T-cell responses in SS through a noncanonical ACSL5-PPARα-MFN2 pathway, which optimizes mitochondrial function and mitochondria-ER contact to support fatty acid oxidation and lipid homeostasis. Our findings suggest that targeting ACSL5 and metabolic pathways may provide a previously unexplored therapeutic strategy for SS and other autoimmune diseases, offering potential translational applications of targeted immunomodulatory therapies.

## Supplementary Material

Supplementary figures and tables.

## Figures and Tables

**Table 1:** The primers used for RT‒qPCR. Related to Methods.

Species	Gene	Forward primer (5'-3')	Reverse primer (5'-3')
*Homo sapiens*	*β-ACTIN*	TTGCCGACAGGATGCAGAA	GCCGATCCACACGGAGT ACT
	*ACSL5*	CCAAGGGGATATTCGGTTGCT	GGCCTCATTTTGTACCTTATCGT
	*FASN*	ACAGCGGGGAATGGGTACT	GACTGGTACAACGAGCGGAT
	*SREBF1*	CGGAACCATCTTGGCAACAGT	CGCTTCTCAATGGCGTTGT
	*ACLY*	ATCGGTTCAAGTATGCTCGGG	GACCAAGTTTTCCACGACGTT
	*ACACA*	CATGCGGTCTATCCGTAGGTG	GTGTGACCATGACAACGAATCT
	*CPT1A*	ATGCGCTACTCCCTGAAAGTG	GTGGCACGACTCATCTTGC
	*PPARA*	TTCGCAATCCATCGGCGAG	CCACAGGATAAGTCACCGAGG
	*PPARG*	ACCAAAGTGCAATCAAAGTGGA	ATGAGGGAGTTGGAAGGCTCT
	*PPARD*	ACTGAGTTCGCCAAGAGCATC	ACGCCATACTTGAGAAGGGTAA
	*ACOX1*	GGAACTCACCTTCGAGGCTTG	TTCCCCTTAGTGATGAGCTGG
	*PNPLA2*	GGCTTCCTCGGCGTCTACTA	TTTACCAGGTTGAAGGAGGGG
	*CCL2*	CAGCCAGATGCAATCAATGCC	TGGAATCCTGAACCCACTTCT
	*CSF1*	AGACCTCGTGCCAAATTACATT	AGGTGTCTCATAGAAAGTTCGGA
	*CSF2*	TCCTGAACCTGAGTAGAGACAC	TGCTGCTTGTAGTGGCTGG
	*IL1B*	TTCGACACATGGGATAACGAGG	TTTTTGCTGTGAGTCCCGGAG
	*TNF*	CCTCTCTCTAATCAGCCCTCTG	GAGGACCTGGGAGTAGATGAG
	*NOS2*	TTCAGTATCACAACCTCAGCAAG	TGGACCTGCAAGTTAAAATCCC
*Mus musculus*	*β-actin*	GGCTGTATTCCCCTCCATCG	CCAGTTGGTAACAATGCCATGT
	*ChIP MFN2 promoter*	TGATCCGGAAAGGAAAACAG	CACCGAAAGGCCACAGTAAT
	*Pcx*	AATGTCCGGCGTCTGGAGTA	ACGCACGAAACACTCGGAT
	*Plpp2*	CATACCGTCCAGACACGATCA	CTCCCAATGAGACAAGGATGAC
	*Coasy*	ATTTCATCACGCACCTCTACAC	GCATAACGCTCTAGCTGTTGTT
	*Acads*	GACTGGCGACGGTTACACA	GGCAAAGTCACGGCATGTC
	*Slc25a1*	TGGAGGCATCGAAATCTGCAT	GTGGGTTCGCTCGTTCATCTA
	*Acsl5*	TTCTCAGACGCCAAGACGTTG	GGCTTTCTGTATCCCAAGCAA
	*Ppara*	TTTCGGCGAACTATTCGGCTG	GGCATTTGTTCCGGTTCTTCTT
	*Pparg*	TTTTCCGAAGAACCATCCGATT	ATGGCATTGTGAGACATCCCC
	*Ppard*	TCCATCGTCAACAAAGACGGG	ACTTGGGCTCAATGATGTCAC
	*Tnf*	CAGGCGGTGCCTATGTCTC	CGATCACCCCGAAGTTCAGTAG
	*Il1b*	TTCAGGCAGGCAGTATCACTC	GAAGGTCCACGGGAAAGACAC
	*Il1a*	GCACCTTACACCTACCAGAGT	AAACTTCTGCCTGACGAGCTT
	*Il17a*	TCAGCGTGTCCAAACACTGAG	CGCCAAGGGAGTTAAAGACTT
	*Ifng*	ATGAACGCTACACACTGCATC	CCATCCTTTTGCCAGTTCCTC
	*Ifnb1*	CTCACCTACAGGGCGGACT	GGCAAAGGCAGTGTAACTCTT
	*Tgfb1*	CCACCTGCAAGACCATCGAC	CTGGCGAGCCTTAGTTTGGAC

**Table 2:** sgRNA sequences used in this study. Related to Methods.

Species	Gene	sgRNA sequences
*Mus musculus*	*Acsl5*	5'-TGTGTACCCGCAGCGCAAGG-3'

**Table 3:** shRNA sequences used in this study. Related to Methods.

Species	Gene	shRNA sequences
*Homo sapiens*	*ACSL5*	5'-TCTCTTGCATAAAGGTTATAA-3'

**Table 4:** siRNA sequences used in this study. Related to Methods.

Species	Gene	siRNA sequences
*Mus musculus*	*Acsl5*	5'-GCUCCUGUCUUUUGCAUAAtt-3' 5'-UUAUGCAAAAGACAGGAGCtt-3'

## Data Availability

The data supporting the findings of this study were obtained from publicly available databases, including the Gene Expression Omnibus (GEO). The datasets analyzed during the current study are available in GEO under the accession numbers GSE23117, GSE127952, GSE40611, and GSE80805. Additional data related to this study, including raw data and supplementary analyses, can be obtained from the corresponding author for reasonal inqury. For any further inquiries related to this study could also contact the corresponding author. No individual participant data or identifiable personal data are included in this study.

## Abbreviations

SS: Sjögren's syndrome
ACSL5: acyl-CoA synthetase long-chain family member 5
FAO: fatty acid oxidation
PPARα: peroxisome proliferator-activated receptor alpha
MFN2: mitofusin 2
MERCs: mitochondria-endoplasmic reticulum contacts
Th1: T helper 1 cells
Th17: T helper 17 cells
Tfh: follicular helper T cells
Treg: regulatory T cells
FA: fatty acid
CPT1A: carnitine palmitoyltransferase 1A
ACSLs: acyl-CoA synthetase long-chain family members
ATP: adenosine triphosphate
MRGs: metabolism-related genes
MSG: minor salivary gland
DEGs: differentially expressed genes
DEMRGs: differentially expressed metabolism-related genes
RIPA: radioimmunoprecipitation assay
PVDF: polyvinylidene fluoride
BSA: bovine serum albumin
HRP: horseradish peroxidase
CCK-8: Cell Counting Kit-8
ELISA: enzyme-linked immunosorbent assay
PFA: paraformaldehyde
H&E: hematoxylin and eosin
LC‒MS/MS: liquid chromatography-tandem mass spectrometry
SD: standard deviation
RNA-seq: RNA sequencing
GO: Gene Ontology
KEGG: Kyoto Encyclopedia of Genes and Genomes
PPAR: peroxisome proliferator-activated receptor
PPI: protein‒protein
