# Supplementary material for: ACSL5 programs fatty acid metabolism and mitochondrial fitness to sustain pathogenic T cells and exacerbate Sjögren's syndrome
Source: Int J Biol Sci. 2026 Jul 13;22(12):6689–708. doi: 10.7150/ijbs.131033 (PMC13411838; doi:10.7150/ijbs.131033)
Supplement: Supplementary file 1 — Supplementary figures and tables. [file ijbsv22p6689s1.pdf]

Supplementary Materials for

ACSL5 Sustains Pathogenic T-Cell Metabolism and Mitochondrial Fitness to Exacerbate Sjögren's Syndrome

Xinyi Ren *et al.*

*Corresponding author: Lingyan Zheng, zhenglingyan@sjtu.edu.cn; Jiayao Fu, fujiayao92@163.com

This PDF file includes:

Supplementary Figures S1 to S7

Supplementary Tables S1 to S2

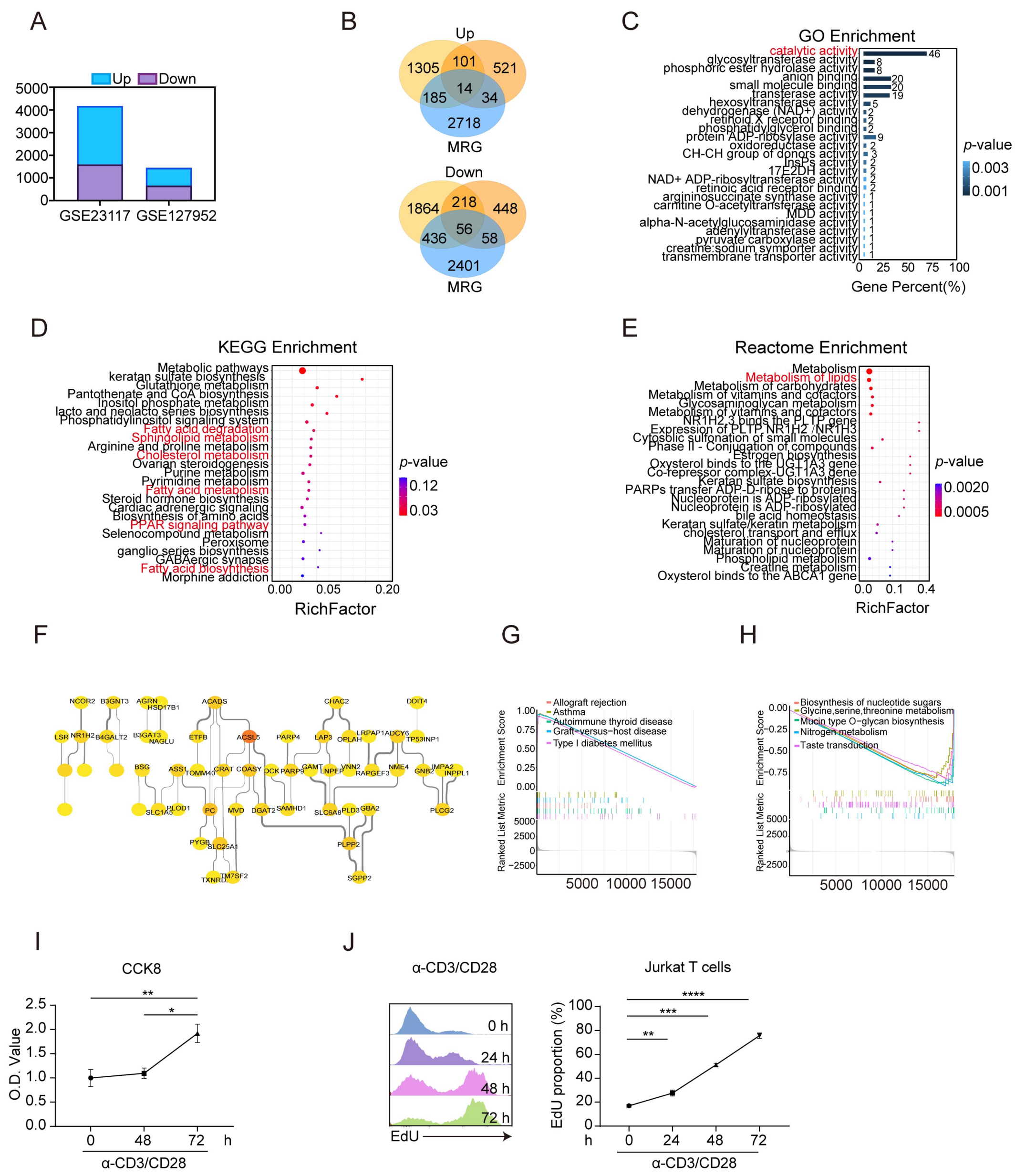

Figure S1 ACSL5 is upregulated upon T-cell activation and SS development. Related to Figure 1 (A) Identification of common DEGs in GSE23117 and GSE127952. (B) Venn diagram of dataset intersection with metabolism gene sets. (C) GO enrichment analysis (molecular function) of MRDEGs. (D) KEGG pathway enrichment analysis of MRDEGs. (E) Reactome enrichment analysis of MRDEGs. (F) PPI network of metabolism-related DEGs. (G) GSEA of the top 5 enriched KEGG pathways for *ACSL5*. (H) GSEA of the top 5 downregulated KEGG pathways for *ACSL5*. (I) Cell viability was determined by a CCK8 assay 72 h after anti-CD3/CD28 stimulation in Jurkat T

cells. (J) Representative EdU flow plots and quantification of proliferating EdU^+ cells of unstimulated vs. stimulated Jurkat T cells. The data are presented as the means ± SDs. n = 3 biological replicates for panels I-J. *$p < 0.05$, **$p < 0.01$, ***$p < 0.001$, ****$p < 0.0001$. Statistical analyses were performed using two-tailed tests, two-way ANOVA, and one-way ANOVA.

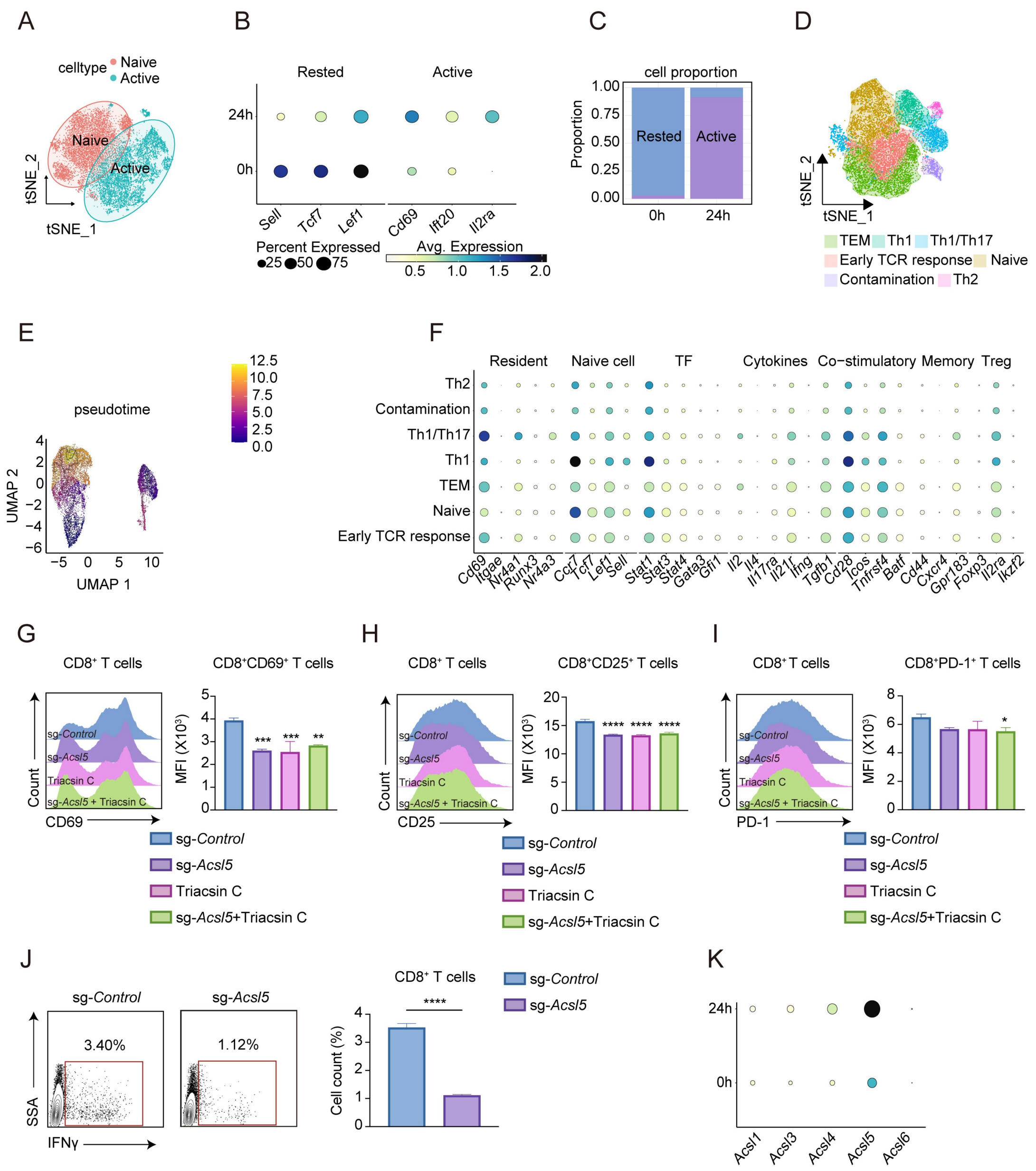

Figure S2 ACSL5 augments CD4^{+} T-cell activation and sustains a proinflammatory phenotype. Related to Figure 2 (A) t-SNE illustration of the scRNA-seq data (GSE179623) comparing unstimulated and T cells stimulated with α-CD3/CD28 after 24 h. (B) Dot plot of T-cell cluster markers in unstimulated (0 h) vs. α-CD3/CD28-stimulated CD4^{+} T cells (24 h). (C) Cell cluster percentages in unstimulated vs. α-CD3/CD28-stimulated CD4^{+} T cells. (D) t-SNE plot of T-cell subtypes in stimulated CD4^{+} T cells. (E) Trajectory analysis of stimulated CD4^{+} T cells. (F) Dot plot of T-cell subtype markers in stimulated CD4^{+} T cells. (G) Flow cytometry of CD69 in CD8^{+}

T cells 72 h poststimulation. (H)Flow cytometric analysis of CD25 in $CD8^+$ T cells at 72 h poststimulation. (I) Flow cytometry analysis of PD-1 expression in $CD8^+$ T cells at 72 h poststimulation. (J) Representative flow cytometric images and quantification of IFN-γ^+ $CD8^+$ T cells 72 h after anti-CD3/CD28 stimulation. (K) Acsl isoform expression in unstimulated vs. α-CD3/CD28-stimulated $CD4^+$ T cells. The data are presented as the means ± SDs. n = 3 biological replicates for panels G-J. *$p < 0.05$, **$p < 0.01$, ***$p < 0.001$, ****$p < 0.0001$. Statistical analyses were performed using two-tailed tests, two-way ANOVA, and one-way ANOVA.

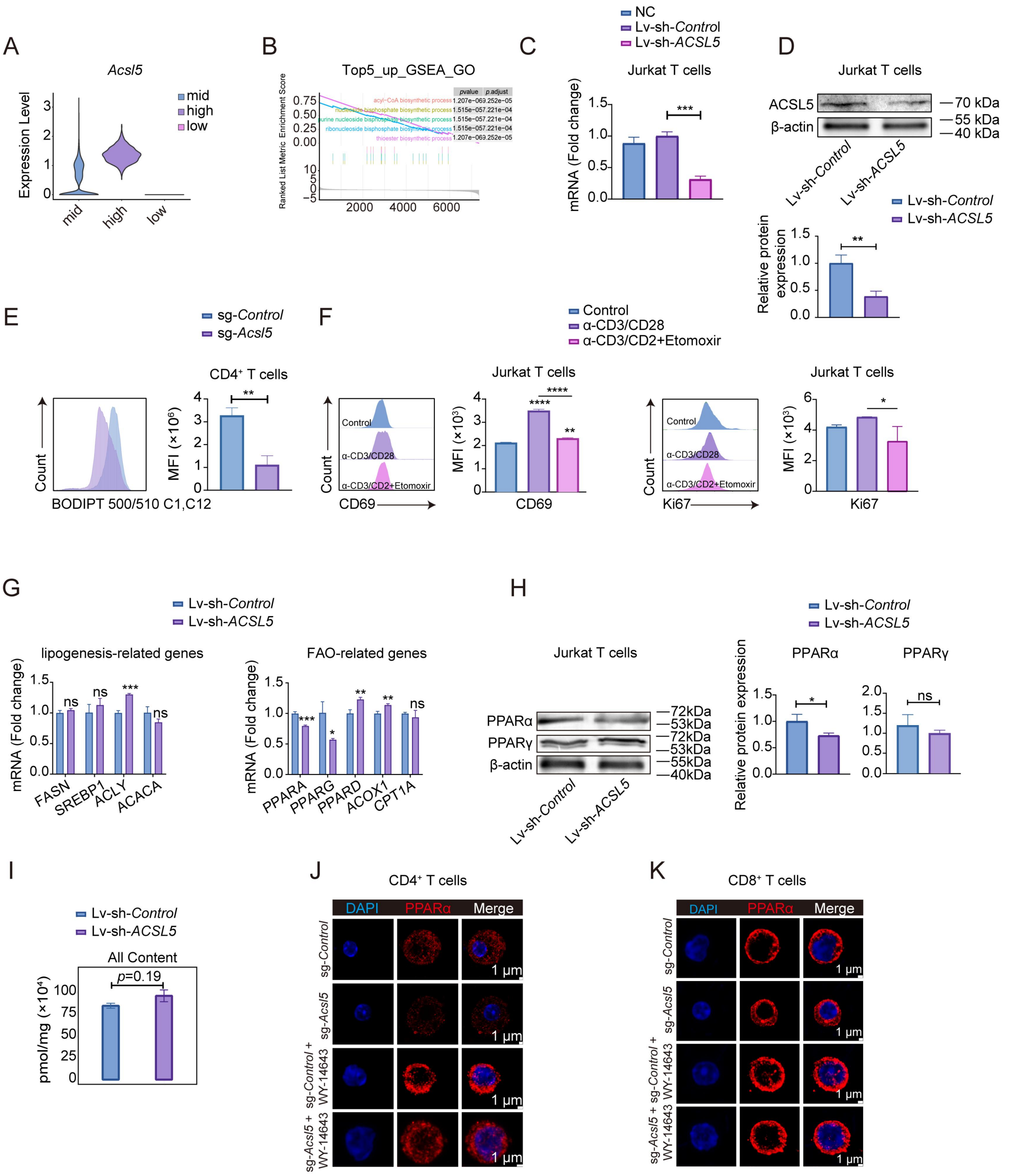

Figure S3 ACSL5 fuels fatty acid oxidation in CD4$^+$ T cells through PPARα. Related to Figure 3 (A) Violin plots showing the expression of ACSL5 divided into high, middle, and low groups. (B) Top 5 enriched GO pathways according to ACSL5 expression level. (C) ACSL5 mRNA expression in Lv-sh-*Control* vs. Lv-sh-*ACSL5* Jurkat T cells. (D) Western blot of ACSL5 in Lv-sh-*Control* vs. Lv-sh-*ACSL5* Jurkat T cells. (E) Lipid uptake (BODIPY C12) in sg-*Control* and sg-*Acsl5* transfected CD4$^+$ T cells. (F) Flow plots and quantification of CD69/Ki67 in stimulated Jurkat T cells ± etomoxir. (G) Lipid metabolism-related gene mRNA levels in Lv-sh-*Control* vs. Lv-sh-*ACSL5* Jurkat T

cells (H) Immunoblotting of PPARα and PPARγ protein levels in Lv-sh-*Control* vs. Lv-sh-*ACSL5* Jurkat T cells. (I) Total lipid content (LC–MS) in Lv-sh-*Control* vs. Lv-sh-*ACSL5* Jurkat T cells. (J) Confocal images of PPARα and DAPI in treated $CD4^+$/$CD8^+$ T cells. PPARα (red), Nuclei (blue). Scale bar = 1 μm. The data are presented as the means ± SDs. n = 3 biological replicates for panels C-K. *p < 0.05, **p < 0.01, ***p < 0.001, ****p < 0.0001. Statistical analyses were performed using two-tailed tests, two-way ANOVA, and one-way ANOVA.

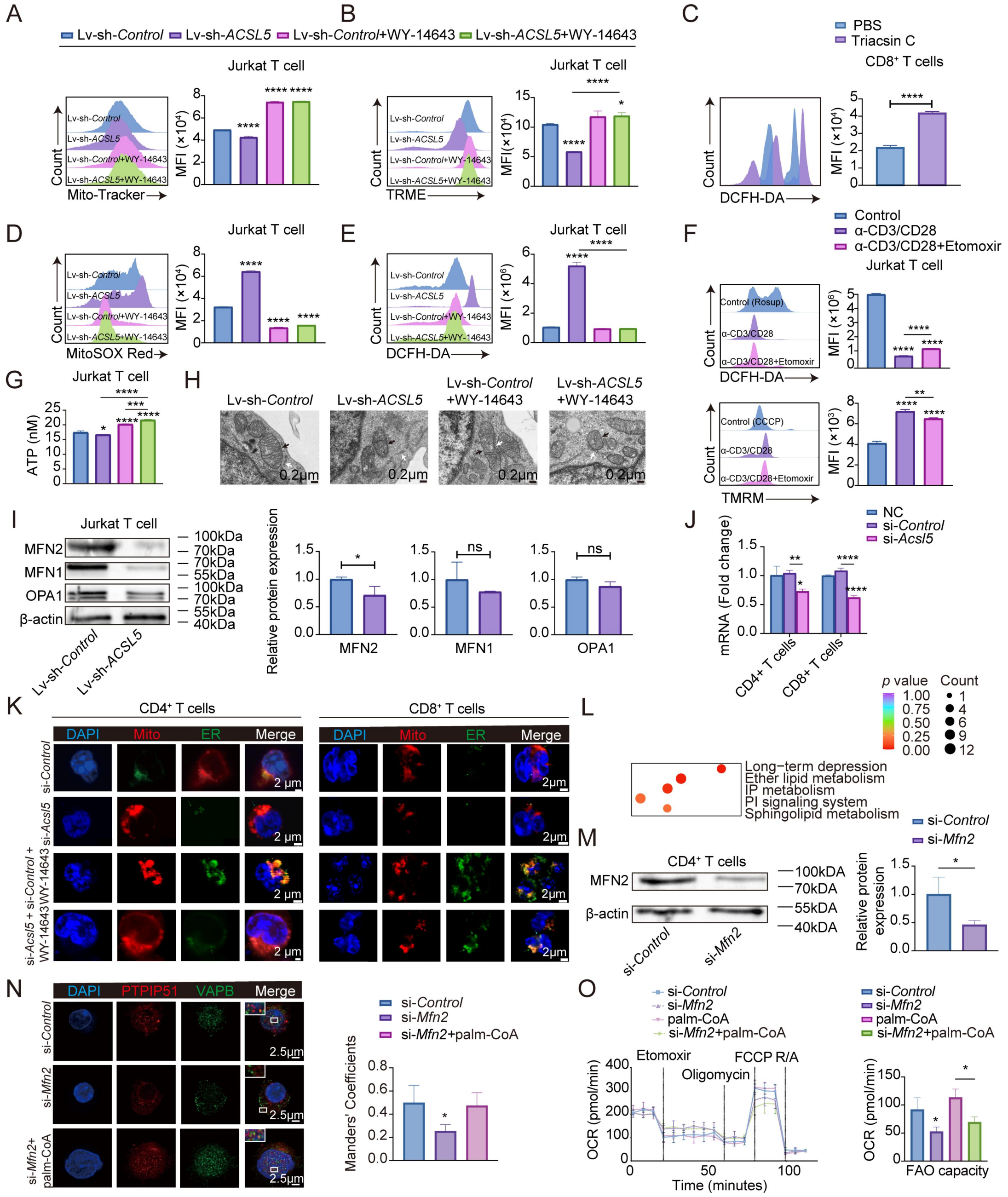

Figure S4 The ACSL5–PPARα axis regulates MFN2-related mitochondrial function to influence FAO. Related to Figure 4 (A) Representative flow cytometry plots of MitoTracker Red staining. (B) Representative flow cytometry plots of TMRE staining. (C) MFI of DCFH-DA in CD8^{+} T cells treated with or without Triacsin C. (D) MitoSox MFI of Lv-sh-Control and Lv-sh-*ACSL5* Jurkat T cells with/without WY-14643. (E) Intracellular ROS levels in Lv-sh-Control and Lv-sh-*ACSL5* Jurkat T cells with/without WY-14643, as determined by flow cytometry. (F) TMRM and DCFH-DA MFI in stimulated Jurkat T cells with/without etomoxir 72 h poststimulation. (G) Intracellular

ATP levels in ACSL5-knockdown and control Jurkat T cells with/without WY-14643. (H) TEM of the mitochondrial structure and MERCs of Lv-sh-*Control* and Lv-sh-*ACSL5* Jurkat T cells with/without WY-14643. Arrows: black (mitochondria), white (ER). Scale bar = 0.2 μm. (I) Western blot of mitochondrial fusion proteins (MFN2, MFN1, and OPA1) in Lv-sh-Control and Lv-sh-*ACSL5* transfected Jurkat cell lines. (J) mRNA levels of Acsl5 in si-*Control* and si-*Acsl5* transfected $CD4^+$ T cells. (K) Confocal microscopy of MitoTracker Red and ER-Tracker Green in si-*Control* and si-*Acsl5* transfected $CD4^+$ and $CD8^+$ T cells. Mitochondria (red), ER (green), Nuclei (blue). Scale bar = 2 μm. (L) Metabolite enrichment analysis in Lv-sh-*Control* and Lv-sh-*ACSL5* Jurkat T cells. (M) Protein levels of MFN2 in si-*Control* and si-*Mfn2* transfected CD4+ T cells. (N) Confocal images of PTPIP51 and VAPB colocalization in $CD4^+$ T cells transfected with si-*Control* or si-*Mfn2* with/without palm-CoA. PTPIP51 (red), VAPB (green), Nuclei (blue). Scale bar = 2.5 μm. Insets show enlarged views of the indicated regions. Colocalization was quantified using Manders' coefficient. (O) Mitochondrial stress test (OCR) of the indicated groups of $CD4^+$ T cells. The data are presented as the means ± SDs. n = 3 biological replicates for panels A-O. *$p < 0.05$, **$p < 0.01$, ***$p < 0.001$, ****$p < 0.0001$. Statistical analyses were performed using two-tailed tests, two-way ANOVA, and one-way ANOVA.

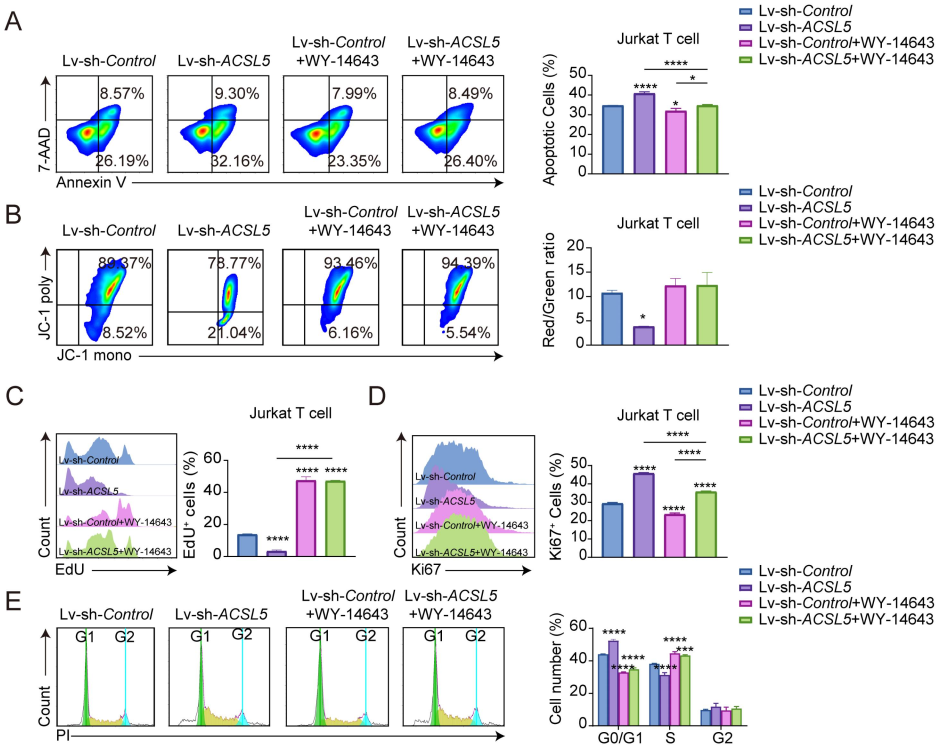

Figure S5 ACSL5/PPARα/FAO-mediated mitochondrial function determines T-cell fate. Related to Figure 5. (A) Apoptosis in Lv-sh-Control and Lv-sh-ACSL5 Jurkat T cells with/without WY-14643. (B) Mitochondrial potential (JC-1) in Lv-sh-Control and Lv-sh-ACSL5 Jurkat T cells with/without WY-14643. (C) EdU proliferation assay of Jurkat T cells. (D) Ki67 proliferation assay of Jurkat T cells. (E) Cell cycle analysis of the indicated cells. The data are presented as the means ± SDs. n = 6 biological replicates for panels F, and n = 3 biological replicates for panels A-E. *$p < 0.05$, **$p < 0.01$, ***$p < 0.001$, ****$p < 0.0001$. Statistical analyses were performed using two-tailed tests, two-way ANOVA, and one-way ANOVA.

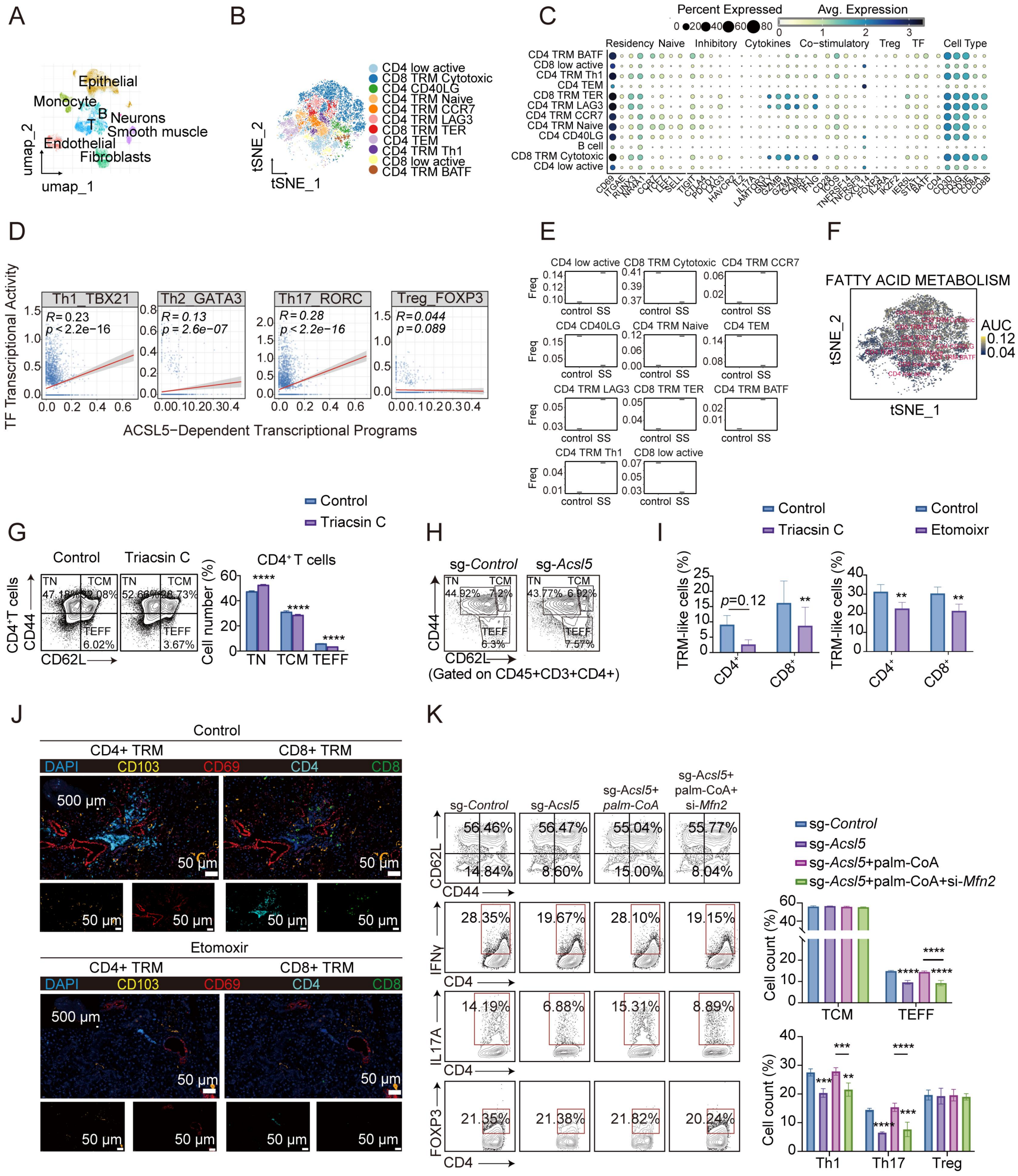

Figure S6 ACSL5/PPARα/FAO-mediated mitochondrial function determines T-cell fate. Related to Figure 6. (A) UMAP plot identifying 8 major clusters. (B) t-SNE visualization of 11 T-cell subsets. (C) Dot plot showing marker expression across annotated cell clusters. (D) Correlations between ACSL5-dependent transcriptional programs and inferred transcription factor (TF) activity across CD4$^+$ T-cell subsets. (E) Violin plots comparing T-cell subclusters in the SS vs. control groups. (F) AUCell scores for selected KEGG pathways in T cells. (G) Flow cytometry analysis of CD44 and CD62L in CD4$^+$ T cells with/without Triacsin (n=3). (H) Exemplary flow

cytometric images of the differentiation of the transferred cells. (I) TRM cell percentages in the SMGs of etomoxir- vs. saline-treated NOD mice (n=5). (J) Immunofluorescence analysis of CD4/8, CD69, and CD103 colocalization in NOD mouse SMGs (scale bar = 50–500 μm). (K) Flow cytometry of $CD4^+$ T-cell subsets in the indicated groups. The data are presented as the means ± SDs. n = 5 biological replicates for panels I-J, and n = 3 biological replicates for panels G-H, K. *p < 0.05, **p < 0.01, ***p < 0.001, ****p < 0.0001. Statistical analyses were performed using two-tailed tests, two-way ANOVA, and one-way ANOVA.

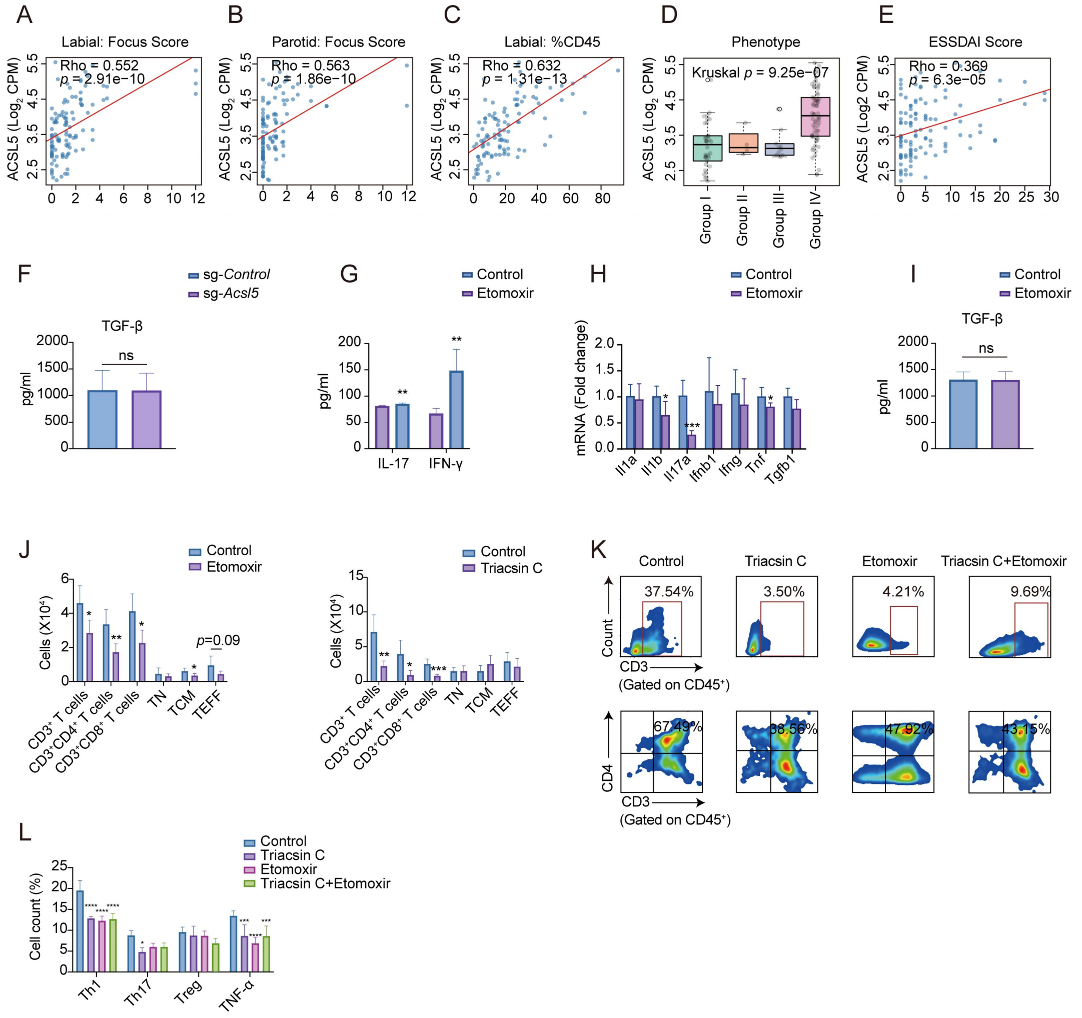

Figure S7 Targeting ACSL5 and FAO reverses SS symptoms *in vivo*. Related to Figure 7. (A) Scatter plots showing positive correlations between ACSL5 expression and focus scores in labial salivary glands. (B) Correlation between ACSL5 levels and focus scores in parotid glands. (C) Positive correlation between ACSL5 levels and the percentage of CD45^{+} immune cell infiltration in labial glands. (D) ACSL5 expression significantly increases with the severity of histological infiltration patterns (Groups I–IV). (E) Correlation of ACSL5 expression with the EULAR Sjögren's Syndrome Disease Activity Index (ESSDAI). (F) Serum TGF-β levels in the indicated groups. (G) Serum IL-17 and IFN-γ levels in Triacsin C- or saline-treated mice at 12 weeks old. (H) Cytokine mRNA levels in the SMGs of Triacsin C- vs. saline-treated NOD mice at 12 weeks old. (I) Serum TGF-β levels in the indicated groups. (J) Total and CD4^{+} T-cell ratios in the SMGs of the indicated groups. (K) Flow cytometry of infiltrating T cells

in Triacsin C/etomoxir-treated vs. control mice at 12 weeks old. (L) T-cell effector function in the indicated groups. The data are presented as the means ± SDs. n = 5 biological replicates for panels F-L. *p < 0.05, **p < 0.01, ***p < 0.001, ****p < 0.0001. Statistical analyses were performed using two-tailed tests, two-way ANOVA, and one-way ANOVA. Correlation analyses were performed using Spearman's rank correlation unless otherwise specified. All correlations shown in (A–E) were calculated using salivary gland RNA-seq data from the GEO dataset GSE173808 (paired labial and parotid glands from 39 SS and 20 non-SS sicca subjects).

Table S1. Antibodies (WB / IF) used in this study. Related to Methods.

TARGET	COMPANY	CATALOG #	HOST
ACSL5	SANTA CRUZ	SC-365230	MOUSE
PPARA	CST	2443	RABBIT
PPARΓ	PROTEINTECH	16643-1-AP	RABBIT
MFN1	PROTEINTECH	13798-1-AP	RABBIT
MFN2	PROTEINTECH	12186-1-AP	RABBIT
OPA1	PROTEINTECH	27733-1-AP	RABBIT
VAPB	ABCAM	AB181869	RABBIT
PTPIP51	ABCAM	AB86018	MOUSE
CD4	SANTA CRUZ	SC-13573	MOUSE
CD8	SANTA CRUZ	SC-7970	MOUSE
CD69	SANTA CRUZ	SC-390889	RABBIT
CD103	SANTA CRUZ	SC-376073	MOUSE
CDK2	CST	2546	RABBIT
CDK4	CST	12790	RABBIT
CYCLIN A2	CST	4656	RABBIT
CYCLIN D3	CST	2936	RABBIT
CASPASE-1	CST	2225	RABBIT
CASPASE-3	CST	9662	RABBIT
CLEAVED CASPASE-3	CST	9664	RABBIT
B-ACTIN	PROTEINTECH	66009-1-IG / 20536-1-AP	MOUSE/RABBIT

Table S2. Antibodies (Flow cytometry) used in this study. Related to Methods.

MARKER	FLUOROPHORE	COMPANY	CATALOG #
CD4	FITC / PE / APC	BIOLEGEND	100406 / 100408 / 100412
CD8	PE / APC / APC-CY7	BIOLEGEND	100708 / 100712 / 100714
CD69	FITC / APC	BIOLEGEND	104506 / 104512
CD25	FITC / APC	BIOLEGEND	102006 / 102012
CD44	FITC / APC / BV421	BIOLEGEND	103006 / 103012 / 103040
CD62L	FITC / APC / PERCP	BIOLEGEND	104406 / 104412 / 104428
CD45	PE-CY7	BIOLEGEND	103114
CD103	PE	BIOLEGEND	121406
PD-1	APC	BIOLEGEND	135210
IFN-Γ	FITC / APC / BV421	BIOLEGEND	505806 / 505810 / 505830
IL-17A	FITC / APC / PERCP-CY5.5	BIOLEGEND	506906 / 506910 / 506914
IL-4	FITC / PE / APC	BIOLEGEND	504104 / 504106 / 504108
FOXP3	APC	BIOLEGEND	126408
